# A reverse vaccinology approach on transmembrane carbonic anhydrases from *Plasmodium* species as vaccine candidates for malaria prevention

**DOI:** 10.1186/s12936-022-04186-7

**Published:** 2022-06-15

**Authors:** Reza Zolfaghari Emameh, Harlan R. Barker, Hannu Turpeinen, Seppo Parkkila, Vesa P. Hytönen

**Affiliations:** 1 Department of Energy and Environmental Biotechnology, National Institute of Genetic Engineering and Biotechnology (NIGEB), 14965/161, Tehran, Iran; 2 Faculty of Medicine and Health Technology, Tampere University, Tampere, Finland; 3 Blueprint Genetics, Helsinki, Finland; 4 Fimlab Laboratories Ltd and Tampere University Hospital, Tampere, Finland

**Keywords:** Reverse vaccinology, Immunoinformatics, Vaccine, Carbonic anhydrase, *Plasmodium* spp., Malaria

## Abstract

**Background:**

Malaria is a significant parasitic infection, and human infection is mediated by mosquito (*Anopheles*) biting and subsequent transmission of protozoa (*Plasmodium*) to the blood. Carbonic anhydrases (CAs) are known to be highly expressed in the midgut and ectoperitrophic space of *Anopheles gambiae*. Transmembrane CAs (tmCAs) in *Plasmodium* may be potential vaccine candidates for the control and prevention of malaria.

**Methods:**

In this study, two groups of transmembrane CAs, including α-CAs and one group of η-CAs were analysed by immunoinformatics and computational biology methods, such as predictions on transmembrane localization of CAs from *Plasmodium* spp., affinity and stability of different HLA classes, antigenicity of tmCA peptides, epitope and proteasomal cleavage of *Plasmodium* tmCAs, accessibility of *Plasmodium* tmCAs MHC-ligands, allergenicity of *Plasmodium* tmCAs, disulfide-bond of *Plasmodium* tmCAs, B cell epitopes of *Plasmodium* tmCAs, and Cell type-specific expression of *Plasmodium* CAs.

**Results:**

Two groups of α-CAs and one group of η-CAs in *Plasmodium* spp. were identified to contain tmCA sequences, having high affinity towards MHCs, high stability, and strong antigenicity. All putative tmCAs were predicted to contain sequences for proteasomal cleavage in antigen presenting cells (APCs).

**Conclusions:**

The predicted results revealed that tmCAs from *Plasmodium* spp. can be potential targets for vaccination against malaria.

**Supplementary Information:**

The online version contains supplementary material available at 10.1186/s12936-022-04186-7.

## Background

*Plasmodium* spp. are parasitic protozoa, the causative agents of malaria in tropical areas, and one of the major causes of worldwide parasitic infection morbidities [1]. Of the 219 million malaria cases reported by World Health Organization (WHO) between 2010 and 2017, the majority were from sub-Saharan Africa (92%) and Southeast Asia (5%), while in 2017, 435,000 deaths from malaria were reported throughout the world [2, 3]. Malaria also causes significant economic burden to society: approximately $US 2.6 billion was invested in 2010 and it is predicted that the cost reaches to $US 6.6 billion by 2020 for prevention, control, and elimination of malaria, the majority of which was invested in African and low-income countries [4]. In addition, nearly $US 4.067 billion was invested in malaria R&D during the period 2010–2016: $US 103 million (3%) in diagnostics, 254 million (6%) in vector control, 934 million (23%) in prevention and vaccines, 1.158 billion (28%) in basic research, 1.465 billion (36%) in treatment and drugs, and 153 million (4%) in unspecified research areas [5].

*Anopheles* species are considered the main vector of malaria infection. Among the approximately 400 *Anopheles* spp., about 70 species, such as *Anopheles aquasalis, Anopheles darlingi, Anopheles gambiae,* and *Anopheles stephensi*, are suggested to be potential vectors of *Plasmodium* [6]. During blood feeding of an infected female *Anopheles* mosquito, the sporozoites of *Plasmodium* are inoculated into the human bloodstream. They migrate from the bloodstream to the liver within 60 min, which is followed by invasion into hepatocytes. After 5.5 days, the sporozoites develop to asexual merozoites (mature schizont of malaria liver stage). The hepatocytes containing mature merozoites (merosomes) burst and each released merozoite is capable of invading and infecting an erythrocyte, where the merozoite develops for 2 days. In the erythrocyte, asexual reproduction results in ~ 16 merozoites, which eventually leads to rupturing of the erythrocyte, releasing of merozoites, and finally invasion of a new series of erythrocytes. The multiplication of merozoites in erythrocytes increases the population of merozoites in the bloodstream 10- to 20-fold every 2 days. In parallel, gametocytes develop in the human blood as precursor sex cells. Subsequent feeding by a mosquito at this stage may take up the gametocytes, and 10 days after ingestion the gametocyte passes maturation stages I–V through the midgut epithelial cells to the basal lamina. This area is the alteration site to form sporozoites. On the 12th day, sporozoites are liberated into the haemocele and migrate to the salivary glands of a female mosquito, transforming the *Plasmodium* parasite into its infectious form [4].

During the asexual erythrocytic stage of malaria the human immune system is activated in a variety of ways in response to various antigens from *Plasmodium*. The humoral response produces antibodies such as immunoglobulin M (IgM), immunoglobulin G1 (IgG1), IgG2, and IgG3. The cellular immunity response produces cytokines, such as IFN-γ, interleukins (ILs) (e.g., IL-1, IL-6, IL-10, IL-12, IL-15, and IL-21), tumour necrosis factor (TNF) [7–11], and chemokines such as monocyte chemoattractant protein-1 (MCP-1), RANTES (regulated upon activation, normal T cell expressed and secreted), monokine-induced by IFN-γ (MIG), and IFN-γ-inducible protein-10 (IP-10) [9]. Both major histocompatibility complex-I (MHC-l) and MHC-II proteins are critical elements orchestrating the functions of T cells, and CD8^+^ T cells-MHC-l and CD4^+^ T cells-MHC-II strongly hamper the development of liver and blood stage in malaria, respectively [12, 13]. Therefore, both cellular and humoral immune responses were evaluated for a malaria vaccine candidate to measure the concentration levels of IgG, IgE, and IgM antibodies in humoral and cytokines such as IL-10, IFN-γ, and TNF in cellular immunological responses [14]. In endemic areas, the concentrations of IgE, IgG, and TNF were higher in children with cerebral malaria than in the control group, while IgE was positively correlated with severity of malaria infection and IgG and TNF were associated with protection against the disease [15].

In past decades, several anti-malaria vaccine studies have been performed using radiation, chemical, and genetically attenuated sporozoites [16–24], recombinant membrane proteins of merozoites [25–30], and synthesized or chimeric antigenic peptides [31–36] as antigens. Although $US 2.7 and 3.1 billion were invested in malaria studies in 2016 and 2017, respectively [37], some important limitations, such as premature vaccine production technologies, short duration of protection, complexity of clinical trial procedures, and inadequate screening in endemic countries, have been identified as the bottlenecks in scaling-up a malaria vaccine [38]. Analysis of the first approved malaria vaccine, Mosquirix or RTS,S/AS01, by the European Medicines Agency (EMA) and WHO, produced a recommendation for vaccination of children aged 6 weeks to 17 months, in sub-Saharan African countries [39, 40]. Phase III clinical trial results of Mosquirix revealed that it was able to induce immunity against *Plasmodium falciparum* infection in 36% of children aged 5–17 months and 26% of infants aged 6–12 weeks [41]. The narrow population age group coverage and low rate of immunity of Mosquirix suggests that other *Plasmodium* proteins or antigens should still be sought as novel targets in vaccine studies.

Carbonic anhydrases (CAs) are encoded by eight evolutionary divergent gene families: α, β, γ, δ, ζ, η, θ, and ι CAs [42–46]. Although all CA families contain a zinc ion in their catalytic active site, certain ζ- and γ-CAs contain cadmium(II), iron(II) or cobalt(II) as alternative metal ion cofactors [47–49]. α-CAs contain 13 catalytically active members in mammals: cytosolic (CA I, CA II, CA III, CA VII, and CA XIII), transmembrane (CA IV, CA IX, CA XII, CA XIV, and CA XV), mitochondrial CAs (VA and CA VB), and secreted CA (VI) [50].

β-CAs are found in plants, algae, fungi, bacteria, and many parasites [43, 51], while they are absent in vertebrate genomes [52, 53]. Previous studies have revealed that CAs play a critical role in midgut pH regulation of *A. gambiae* through anion transport [54], and in detoxification of cyanate in *Ascaris lumbricoides* by providing bicarbonate for the activity of cyanase [55]. They are, therefore, considered potential target enzyme candidates for novel anti-infectives [43, 55–60]. Antigenic site prediction of parasite β-CAs has determined that the second highly conserved sequence motif (HXXC; H: Histidine, C: Cysteine; and X: any amino acid) is the most antigenic site of the protein. However, a protein modelling study identified that this conserved sequence was buried in relation to the surface of the protein and, therefore, the accessibility of the epitope to the human immune system would be far from optimal [57].

The first identified η-CA was found in *Plasmodium yoelii*, and was studied as a potential drug target for inhibition and treatment of malaria in 2014 [42]. In 2016, a study of *P. falciparum* revealed that η-CA plays a crucial role in the pyrimidine biosynthetic pathway, via preparation of the HCO_3_^−^ needed for expression of six pyrimidines, including: carbamoylphosphate synthetase II (CPSII) and aspartate transcarbamoylase (ATC), dihydroorotase (DHO), dihydroorotate dehydrogenase (DHOD), orotate phosphoribosyltransferase (OPRT), and orotidine 5′-monophosphate decarboxylase (OMPDC) [61]. Due to the surface accessibility of *Plasmodium* transmembrane CAs (tmCAs) to the human immune system, they are studied here to find alternative antigens or peptide sequences for development of antimalaria vaccines.

In this study, *Plasmodium* tmCAs were analysed using reverse vaccinology and immunoinformatic approaches. With these methods, the most antigenic sites of *Plasmodium* tmCAs were identified, which could possess the highest binding affinity to MHCs and B cell epitopes.

## Methods

### Identification of *Plasmodium* tmCAs

The *Plasmodium* tmCA protein sequences were collected from the UniProt database (http://www.uniprot.org/) [62] used them to perform protein homology BLAST searches using the EMBL-EBI BLAST database (http://www.ebi.ac.uk/Tools/sss/wublast/) [63]. To differentiate and categorize the BLAST results, they were aligned with the *Plasmodium* tmCA protein sequences using Clustal Omega (http://www.ebi.ac.uk/Tools/msa/clustalo/) [64]. In addition, *Plasmodium* and membrane-bound human (*Homo sapiens*) α-CA (hCAs) protein sequences were aligned, including CA4 (P22748), CA9 (Q16790), CA12 (O43570), and CA14 (Q9ULX7). Protein models in the Protein data bank (PDB) (http://www.rcsb.org/pdb/home/home.do) [65] and structural biology knowledgebase (SBKB) (http://sbkb.org/) [66] were used to investigate the structural similarity between the *Plasmodium* tmCAs and hCAs.

### Prediction of transmembrane localization of CAs from *Plasmodium* spp.

Prediction of transmembrane helices of *P. falciparum* α-CAs (W7JAI7 and Q8IHW5 from group 1 and group 2, respectively) and the *P. yoelii* η-CA (V7PFH4), was performed using TMHMM Server v. 2.0 (http://www.cbs.dtu.dk/services/TMHMM/) [67]. TMHMM has been shown to accurately predict 97–98% of transmembrane domains and can also differentiate between membrane and soluble proteins with 99% sensitivity and specificity [67].

### Prediction of affinity and stability of different HLA classes

Affinity to MHC and tmCAs-MHC complex stability predictions for different HLA molecules from MHC-l were analysed using NetMHCpan 2.8 (http://www.cbs.dtu.dk/services/NetMHCpan/) [68, 69] and NetMHCstab 1.0 (http://www.cbs.dtu.dk/services/NetMHCstab-1.0/) [70] servers. NetMHCpan is the most complete and comprehensive prediction server for MHC binding prediction and is based on quantitative MHC-binding data, which covers binding of human HLA-A and HLA-B to protein sequences. NetMHCpan is able to discriminate between stable and unstable peptide binders within the protein sequences. Prediction of peptide-MHC complex stability and affinity of various MHC-II HLA antigens to *Plasmodium* tmCAs were studied using the NetMHCIIpan 3.1 Server (http://www.cbs.dtu.dk/services/NetMHCIIpan/) [71]. This database provides a quantitative tool to predict binding of known human protein sequences to MHC-II.

### Prediction of antigenicity of tmCA peptides

To locate antigenic sites on tmCAs, the European Molecular Biology Open Software Suite (EMBOSS) antigenic tool (http://emboss.bioinformatics.nl/cgi-bin/emboss/antigenic) [72] was used. This tool is based on the Kolaskar and Tongaonkar method for detection of antigenic sites of protein sequences. The analysis was performed for group 1 (W7JAI7) and 2 (Q8IHW5) of the α-CAs and η-CA (V7PFH4) from *P. falciparum* and *P. yoelii*, respectively.

### Epitope prediction and proteasomal cleavage of *Plasmodium* tmCAs

The prediction of epitopes for both MHC-I and MHC-II in representative *P. falciparum* tmCAs, from group 1 (W7JAI7) and 2 (Q8IHW5), and the *P. yoelii* η-CA (V7PFH4), was performed using the Immune Epitope Database and Analysis Resource (IEDB) (http://www.iedb.org/home_v3.php) [73]. Peptide sequences having high MHC-binding affinity, MHC-peptide complex stability, and high antigenicity were analysed for epitope prediction.

Proteasomes play a crucial role in the human immune response. Antigens are processed by proteasomes in the antigen presenting cells (APCs) and are then presented at the surface of APCs in complex with MHC-I [7]. Proteasomal cleavage analysis of *Plasmodium* tmCAs was conducted using the Proteasomal Cleavage Prediction Reference—IEDB Analysis Resource (http://tools.iedb.org/processing/) [73, 74].

### Accessibility of predicted *Plasmodium* tmCAs MHC-ligands

In order to analyse the accessibility of antigens on protein 3D structures, BLAST search within the Protein Data Bank (PDB) (http://www.rcsb.org/pdb/home/home.do) was used to identify those structures with the greatest similarity to the proteins of interest [75]. The corresponding sequences were then used to prepare a multiple sequence alignment (MSA) using the Clustal Omega algorithm (http://www.ebi.ac.uk/Tools/msa/clustalo/) [64]. The peptide sequences were then mapped on the 3d structures using the alignments. The protein structures retrieved from PDB together with the sequence alignment was used to generate structural superpositions using PyMOL 0.99 [76]. Cartoon models and surface representations were rendered using VMD 1.9.1 [77] and edited using Gimp 2.8.14 [78] Sequence similarity was calculated using Ident and Sim (https://www.bioinformatics.org/sms2/ident_sim.html).

### Allergenicity prediction of *Plasmodium* tmCAs

The allergenicity of representative tmCAs (W7JAI7, Q8IHW5, and V7PFH4) from *Plasmodium* was analysed using the SDAP (Structural Database of Allergenic Proteins) database (https://fermi.utmb.edu/) [79, 80]. This allowed identification of allergens from the IUIS (International Union of Immunological Societies) (http://www.allergen.org) with at least 35% identity [81] with tmCAs, based on FAO/WHO (Food and Agriculture Organization of the United Nations/World Health Organization). In this database, the allergenicity prediction is performed using an MSA of full-length tmCAs with a sliding window of 80 amino acids (80mer).

### Disulfide-bond prediction of *Plasmodium* tmCAs

Prediction of *Plasmodium* tmCAs disulfide-bonds (W7JAI7, Q8IHW5, and V7PFH4) was performed using DISULFIND (http://disulfind.dsi.unifi.it/) [82]. DISULFIND predicts the disulfide-bond connectivity of cysteine (Cys) residues along protein sequences [83, 84]. The disulfide-bonds plays a critical role in intramolecular stability of the proteins, especially the candidate proteins for vaccine design.

### Prediction of B cell epitopes of *Plasmodium* tmCAs

Prediction of linear B cell epitopes in *Plasmodium* tmCAs (W7JAI7, Q8IHW5, and V7PFH4) was performed using the IEDB Analysis Resource (http://tools.iedb.org/bcell/) [85], using all settings as default. In this analysis, the predicted epitopes of Plasmodium tmCAs against MHC-I and MHC-II were checked for any potential similarity with the predicted B cell epitopes obtained in this analysis.

### Cell type-specific expression of *Plasmodium* CAs

RNA sequencing (RNA-Seq) expression data for plasmodium in various stages of the life cycle were retrieved from the "malaria.tools" webserver (https://malaria.sbs.ntu.edu.sg) (collected 2019–06-27) [86]. Expression data for carbonic anhydrase PBANKA_0909000 was available for 12 samples in 5 lifecycle stages in *Plasmodium berghei*; similarly, for *P. falciparum* carbonic anhydrase PF3D7_1140000 data were available for 42 samples in 4 lifecycle stages. Expression values were plotted using the Seaborn (https://zenodo.org/record/3767070) (PMID: 29409532) and Matplotlib (https://doi.org/10.1109/MCSE.2007.55) Python graphical libraries.

## Results

### Identification of *Plasmodium* tmCAs

19 *Plasmodium* tmCA protein sequences were collected from the UniProt database. Protein homology BLAST search revealed that all the tmCAs belonged to α- or η-CA families. Based on conservation of the three histidines characteristic of α-CA catalytic active sites, and the level of protein sequence homology, *Plasmodium* α-CAs were categorized to two groups, 1 and 2 (Additional file 1: Fig. S1 and Fig. S2). Based on this categorization, one putative enzyme was selected from each group as a representative sample for further analysis; W7JAI7 from group 1 and Q8IHW5 from group 2, both of which are CAs from *P. falciparum*. The amino acid composition of the *Plasmodium* η-CA catalytic active site, which contained four histidines and one phenylalanine, was different from that of the α-CAs (Additional file 1: Fig. S3). Multiple sequence alignment (MSA) revealed considerable differences between group 1 and group 2 *Plasmodium* and human transmembrane α-CAs (Additional file 1: Fig. S4 and Fig. S5). Moreover, MSA results showed significant differences between human α-CAs and *Plasmodium* η-CAs (Additional file 1: Fig. S6). Utilizing the structural biology knowledgebase (SBKB) database, W7JAI7 was identified showing the highest similarity (28%, E = 2.1E-4) to hCA-II (PDB ID: 4HBA) [87], which should be acceptable considering the use of *Plasmodium* CAs for vaccination [61].

### CAs from *Plasmodium* are predicted to be transmembrane proteins

Cellular localization predictions for the representative α-CAs from group 1 (W7JAI7) and group 2 (Q8IHW5) of *P. falciparum* and the η-CA (V7PFH4) of *P. yoelii* revealed that each contains a single transmembrane helix in the C-terminal region, including amino acid residues 501–523 for W7JAI7, 577–599 for Q8IHW5, and 669–686 for V7PFH4. The N-termini of these proteins were predicted as extracellular, while at the C-terminal ends, 1–3 terminal amino acid residues were predicted as intracellular (Additional file 1: Fig. S7).

### Prediction of MHC-binding peptides within *Plasmodium* tmCAs

The prediction defined that three extracellular peptide sequences of W7JAI7 (group 1) and three extracellular peptide sequences of Q8IHW5 (group 2) from α-CAs have high predicted binding affinity to the human leukocyte antigen-A*11 (HLA-A*11), HLA-A*24, and HLA-B*53 alleles of human MHC-l (Table 1) and, therefore, the potential to elicit humoral immune response and provide immunological memory. Prediction of antigenicity, using the artificial neural network methods of the NetMHCpan and NetMHCstab servers, identified peptides which were predicted to bind strongly to MHCs. The prediction analysis for the η-CA (V7PFH4) from *P. yoelii* displayed high MHC-I binding affinity (HLA-A*02 and HLA-B*53 alleles), high peptide-MHC-I complex stability, and strong antigenicity of two extracellular peptide sequences (Table 1).

**Table 1:** Prediction of binding affinity and MHC-peptide complex stability for α- and η-CA protein sequences from *Plasmodium* spp.

CA class	HLA-A and -B alleles	Peptide sequence	NetMHCpan Affinity^a^ (0 ≤ X ≤ 1)	NetMHCstab Stability (hours)	Antigenicity prediction	Result^b^
α-CA (group 1) (W7JAI7)	HLA-A*24	509-IYFILFIFYNIVLF-522	0.913	15.14	501-FSYYSKWDIYFILFIFYNIVL-521 (Transmembrane sequence)	HS
	HLA-A*11	399-STLPLCDENVSWK-411	0.899	13.09	398-SSTLPLCDEN-407	HS
	HLA-B*53	469-FPIQVLISSAI-479	0.717	–	461-RKFSLVQVFPIQVLISSAIS-480	SB
α-CA (group 2) (Q8IHW5)	HLA-A*24	585-IYFILFIFYNIVLF-598	0.913	15.14	582-KWDIYFILFIFYNIVL-597 (Transmembrane sequence)	HS
	HLA-A*11	476-STLPLCDENVSWK-488	0.899	13.09	475-SSTLPLCDEN-484	HS
	HLA-B*53	8-YPILLFYNVNVF-19	0.695	–	4-LYLLYPILLFYNVNVFINY-22	SB
η-CA (V7PFH4)	HLA-A*02	633-YLIQGFPV-640	0.925	17.67	633-YLIQGFPV-640	HS
	HLA-B*53	638-FPVQLLISSAL-648	0.638	–	629-YGRVYLIQGFPVQLLISSALT-649	SB

*HS* highly stable, *SB* strong binding

Transmembrane domains of α-CAs (W7JAI7 and Q8IHW5) from *P. falciparum*, which are suggested to be buried from the access of MHC-I and MHC-II systems

^a^Threshold for SB is 0.5%

^b^The threshold stability for HS and SB is 6 h. Strong binding peptides (IC_50_ ≤ 50 nM or %-Rank ≤ 0.5)

High affinity, stability, and antigenicity to human MHC-II (including MHC-II subtype DRB1), were predicted for two extracellular peptide sequences of W7JAI7 (group 1) and one extracellular peptide sequences of Q8IHW5 (group 2), both of which are *P. falciparum* α-CAs. Only one extracellular peptide sequence of the η-CA (V7PFH4) from *P. yoelii* displayed high affinity, stability, and antigenicity to the human MHC-II molecule DQ (Table 2).

**Table 2:** Prediction of affinity of different HLAs of MHC-II alleles against top domains of α- and η-CA protein sequences from *Plasmodium* spp.

CA class	MHC-II type	Peptide sequence	Affinity (0 ≤ X ≤ 1)	Antigenicity prediction	Result^d^
α-CA^a^ (group 1) (W7JAI7)	DRB1	428-LRTIINVSSAVHVGS-442	0.811	428-LRTIINVSSAVHVGSDPTLVELK-450	SB
		468-VFPIQVLISSAISNI-482	0.873	461-RKFSLVQVFPIQVLISSAIS-480	SB
α-CA^b^ (group 2) (Q8IHW5)	DRB1	424-SEKFLRTIINVSSAV-438	0.732	428-LRTIINVSSAVHVGSDPTLVELK-450	SB
η-CA^c^ (V7PFH4)	DQ	629-YGRVYLIQGFPVQLL-643	0.544	629-YGRVYLIQGFPVQLLISSALT-649	SB

^a^MHC II HLA-DRB3, -DRB4, -DRB5, -DP, and -DQ did not show any affinity to group 1 (W7JAI7) of α-CA from *P. falciparum*

^b^MHC II HLA-DRB3, -DRB4, -DRB5, -DP, and -DQ did not show any affinity to group 2 (Q8IHW5) of α-CA from *P. falciparum*

^c^MHC II HLA-DRB1, -DRB3, -DRB4, -DRB5, and -DP did not show any affinity to η-CA (V7PFH4) from *P. yoelii*

^*d*^*SB* strong binding peptides (the default value is IC_50_ ≤ 50 nM or %-Rank ≤ 0.5). IC_50_ values in nM and %-Rank are the results after the prediction using NetMHCpan 2.8 [100]

### Epitope prediction and proteasomal cleavage of *Plasmodium* tmCAs

Epitope prediction of group 1 (W7JAI7) and 2 (Q8IHW5) of α-CAs from *P. falciparum* and η-CA (V7PFH4) from *P. yoelii* for both MHC-I (Table 3) and MHC-II (Table 4) suggested that the peptide sequences with high affinity to MHC-I and -II obtained high scores among all predicted epitopes.

**Table 3:** Predicted epitopes of *Plasmodium* tmCAs against MHC-I

CA class	HLA-A and -B alleles	Peptide sequence	Predicted epitope for MHC-I	Score^a^
α-CA (group 1) (W7JAI7)	HLA-A*24	509-IYFILFIFYNIVLF-522	509-IYFILFIFYNIVLF-522	0.1
	HLA-A*11	399-STLPLCDENVSWK-411	399-STLPLCDENVSWK-411	0.3
	HLA-B*53	469-FPIQVLISSAI-479	469-FPIQVLISSAISNI-482	0.2
α-CA (group 2) (Q8IHW5)	HLA-A*24	585-IYFILFIFYNIVLF-598	585-IYFILFIFYNIVLF-598	0.1
	HLA-A*11	476-STLPLCDENVSWK-488	476-STLPLCDENVSWK-488	0.3
	HLA-B*53	8-YPILLFYNVNVF-19	8-YPILLFYNVNVF-19	0.2
η-CA (V7PFH4)	HLA-A*02	633-YLIQGFPV-640	633- YLIQGFPVQLLI-644	0.2
	HLA-B*53	638-FPVQLLISSAL-648	638- FPVQLLISSALTT-650	0.5

^a^Low percentile rank = good binders (Range limit: 0.0–100.0)

**Table 4:** Predicted epitopes of *Plasmodium* tmCAs against MHC-II

CA class	MHC-II type	HLA allele	Peptide sequence	Predicted epitope for MHC-II	Score^a^
α-CA* (group 1) (W7JAI7)	DRB1	HLA-DRB1*01:03	428-LRTIINVSSAVHVGS-442	428-LRTIINVSSAVHVGS-442	0.06
			468-VFPIQVLISSAISNI-482	468-VFPIQVLISSAISNI-482	0.19
α-CA** (group 2) (Q8IHW5)	DRB1	HLA-DRB1*01:02	424-SEKFLRTIINVSSAV-438	424-SEKFLRTIINVSSAV-438	0.86
η-CA*** (V7PFH4)	DQ	HLA-DQA1*01:01/DQB1*05:01	629-YGRVYLIQGFPVQLL-643	629-YGRVYLIQGFPVQLL-643	1.59

^a^Low percentile rank = good binders (Range limit: 0.0–100.0)

Antigenic peptides may be produced as a result of proteosomal cleavage, but the cleavage may also destroy the epitopes [88]. Proteasomal cleavage analysis of *Plasmodium* tmCAs revealed that some of the peptide sequences which have good antigenicity and high affinity and stability with MHC-I, consequently had high proteasome cleavage scores (Table 5).

**Table 5:** Proteasomal cleavage of α- and η-CAs from *Plasmodium* spp.

CA class	HLA-A and -B alleles	Proteasome cleaved peptides	Score^a^
α-CA (group 1) (W7JAI7)	HLA-A*24	509-IYFILFIFYNIVLF-522	1.25
	HLA-A*11	399-STLPLCDENVSWKV-412	1.04
	HLA-B*53	467-QVFPIQVLISSAIS-480	1.18
α-CA (group 2) (Q8IHW5)	HLA-A*24	585-IYFILFIFYNIVLF-598	1.25
	HLA-A*11	476-STLPLCDENVSWKV-489	1.04
	HLA-B*53	8-YPILLFYNVNVF-19	1.41
η-CA (V7PFH4)	HLA-A*02	632-VYLIQGFPVQL-642	1.62
	HLA-B*53	638-FPVQLLISSAL-648	1.42

^a^High value predicts high cleavage efficiency (Range limit: 0.0–2.0)

### Accessibility of the predicted MHC-ligands in *Plasmodium* tmCAs

For each of the peptide sequences predicted to bind MHC, the study attempted to identify known CA protein structures in the PDB database with homologous sequence and then used the known structures to ascertain surface placement and therefore MHC accessibility of the peptides.

#### MHC-I predicted ligands, group 1 (W7JAI7)

**Fig. 1:**
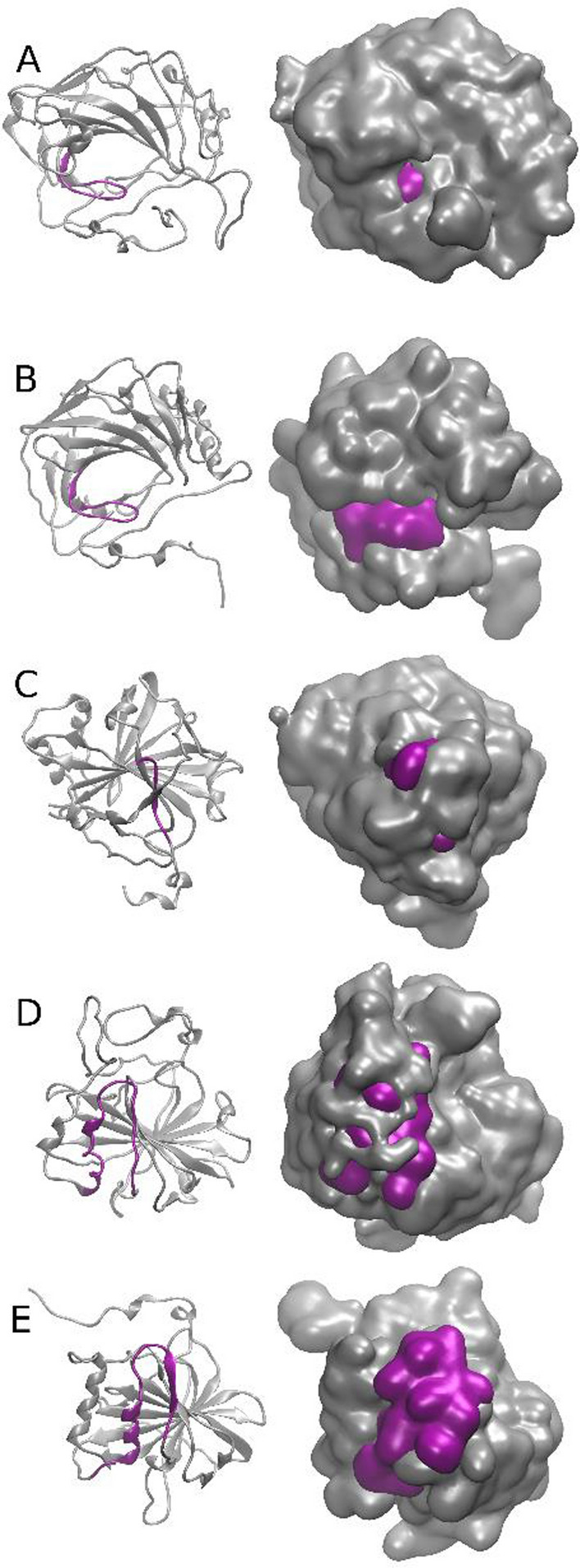
**A**–**E** Accessibility of the predicted *Plasmodium* tmCAs MHC epitopes in homologous PDB models. **A** A sequence homologous to peptide “461-RKFSLVQVFPIQVLISS-477″ in PDB 2W2J is shown in purple. **B** Sequence homologous to "SSTLPLCDENVSWK" in PDB model 4XIW. **C** The sequence homologous to “YGRVYLIQGFPVQLLISSALT” in PDB model 3FE4 (only domain of the sequence covered by the structure). **D** The sequence homologous to”428-LRTIINVSSAVHVGSDPTLVELK-450″ in PDB model 2W2J. **E** The sequence homologous to predicted MHC-II ligand “SEKFLRTIINVSSAV” is highly accessible in PDB 4XIW

BLAST search of the PDB database revealed PDB model 2W2J to have the highest similarity for the protein of interest. The MSA analysis (Additional file 1: Fig. S8) revealed that the C-terminal peptide "461-RKFSLVQVFPIQVLISSAISNIEDKKVINIIKDISPKNMSFSYYSKWDIYFILFIFYNIVFLF-524" of W7JAI7 would be located on the C-terminal end of the 2W2J resolved crystal structure, but however is not a part of the described protein. To further evaluate this 63 AA sequence, a BLAST search of the PDB database was performed which revealed no similarity to any known protein structures. Therefore, it may be considered that the C-terminal sequence “461-RKFSLVQVFPIQVLISS-477” to be well accessible and to act as a flexible structure. The peptide “STLPLCDENVSWK” has high similarity (61.5%) with the corresponding segment in the known protein structure PDB 2W2J and it appears to correspond to a rather accessible loop (Fig. 1A).

#### MHC-I predicted ligands, group 2 (Q8IHW5)

N-terminal green sequence “LYLLYPILLFYNVNVFINY” is located in the vicinity of the secondary structure elements of the X-ray structures of several similar CA proteins and can be thus proposed to be accessible (Additional file 1: Fig. S9). Sequence 50.0% similar to “SSTLPLCDENVSWK” can be located in a homologous protein (PDB ID: 4XIW) (Fig. 1B) and the location of the peptide in that protein is virtually identical to the “STLPLCDENVSWK” observed in Group 1. Based on the structural inspection (Fig. 1B), the peptide is predicted to be well accessible to solvent.

#### MHC-I predicted ligands, η-CA (V7PFH4)

The peptide “YGRVYLIQGFPVQLLISSALT” had only low similarity (19.1%) to the corresponding region of the homologous protein PDB 3FE4. The sequence for this structure ends with **“FPN”** corresponding to **“FPV”** within the target peptide, and thus the C-terminal part of the target peptide is not present in the homologous CA structure and might therefore represent a flexible segment (Fig. 1C and Additional file 1: Fig. S10).

#### MHC-II predicted ligands, group 1 (W7JAI7)

C-terminal sequence “461-RKFSLVQVFPIQVLISSAISNI-482″ cannot be assigned to any known protein domains and could therefore represent non-structural, well-accessible segment. The sequence”428-LRTIINVSSAVHVGSDPTLVELK-450″ can be assigned to the protein structure PDB 2W2J (similarity 37.5%) and it appears to correspond to solvent-accessible loop (Fig. 1D and Additional file 1: Fig. S11).

#### MHC-II predicted ligands, group 2 (Q8IHW5)

The predicted MHC-II ligand “SEKFLRTIINVSSAV” has 53.3% sequence similarity with the corresponding segment in a homologous protein (PDB 4XIW; Fig. 1E and Additional file 1: Fig. S12) and is predicted to be accessible.

#### MHC-II predicted ligands, η-CA (V7PFH4)

The predicted MHC-II ligand is accessible and is much overlapping with that shown in Fig. 1C and Additional file 1: Fig. S13.

### Allergenicity prediction of *Plasmodium* tmCAs

Allergenicity was predicted using SDAP (Structural Database of Allergenic Proteins) database (https://fermi.utmb.edu/) (79, 80) for tmCAs W7JAI7, Q8IHW5, and V7PFH4 from *Plasmodium* (Table 6). For W7JAI7 from α-CA group 1, similarity was found to some insect and nematode allergens; for Q8IHW5 from α-CA group 2, similarity was found to a food allergen; and for the η-CA V7PFH4, similarity was found to one insect and multiple food allergens. None of the recorded protein sequence identities were greater than 35%, when using an 80mer sliding window.

**Table 6:** Allergenicity prediction of *Plasmodium* tmCAs to closest identified allergens

CA class	Allergen	Protein name of allergen	Species	Common name of species	UniProt ID	Maximum identity (%)
α-CA (group 1) (W7JAI7)	Vesp c 5	Venom allergen 5.02	*Vespa crabo*	European hornet (insect)	P35782	27.50
	Aed a 1	Apyrase	*Aedes aegyptii*	Malaria mosquito (insect)	P50635	27.50
	Tab y 1.0101	Apyrase	*Tabanus yao*	Horsefly(insect)	B3A0N5	27.50
	Ani s 2	Paramyosin	*Anisakis simplex*	Anisakiasis agent (nematode)	Q9NJA9	27.50
α-CA (group 2) (Q8IHW5)	Len c 1.0102	Vicilin	*Lens culinaris*	Lentil (food)	Q84UI0	28.75
η-CA (V7PFH4)	For t 1.0101	Serine/threonine protein kinase	*Forcipomyia taiwana*	Biting midge (insect)	B2ZPG6	28.75
	Tri a gliadin	Gliadin	*Triticum aestivum*	wheat (food)	A5JTR6	28.75
	Cra g 1	Tropomyosin	*Crassostrea gigas*	Pacific oyster (food)	Q95WY0	28.75
	Pru du 6	Amandin (prunin)	*Prunus dulcis*	Almond (food)	Q43607	28.75
	Jug r 4.0101	11S globulin seed storage protein	*Juglans regia*	English walnut (food)	Q2TPW5	28.75

### Disulfide-bond prediction of *Plasmodium* tmCAs

Disulfide-bond prediction study of Cys residues in the tmCAs (W7JAI7, Q8IHW5, and V7PFH4) from *Plasmodium* determined that Cys122 and Cys405 from W7JAI7 (α-CA, group 1) and Cys198 and Cys481 from Q8IHW5 (α-CA, group 2) are involved in formation of disulfide-bonds. The results revealed two disulfide-bonds between Cys12-Cys14 and Cys281-Cys574 in V7PFH4 (η-CA). The confidence of connectivity (Conn_conf) was calculated to be 1 for W7JAI7 and Q8IHW5, and 0.8 for V7PFH4 (Additional file 1: Table S1). The prediction inspected the crystal structures of homologous proteins, which revealed that residues corresponding to Cys122 and Cys405 in W7JAI7 are in close proximity in PDB 2W2J but only the residue corresponding to Cys405 is conserved. Residues corresponding to Cys198 and Cys481 from Q8IHW5 form a disulfide in homologous structure PDB 4XIW. Evaluation of the predicted disulfides in V7PFH4 was not possible due to poor sequence coverage of the homologous crystallized proteins.

### Prediction of B cell epitopes in *Plasmodium* tmCAs

**Fig. 2:**
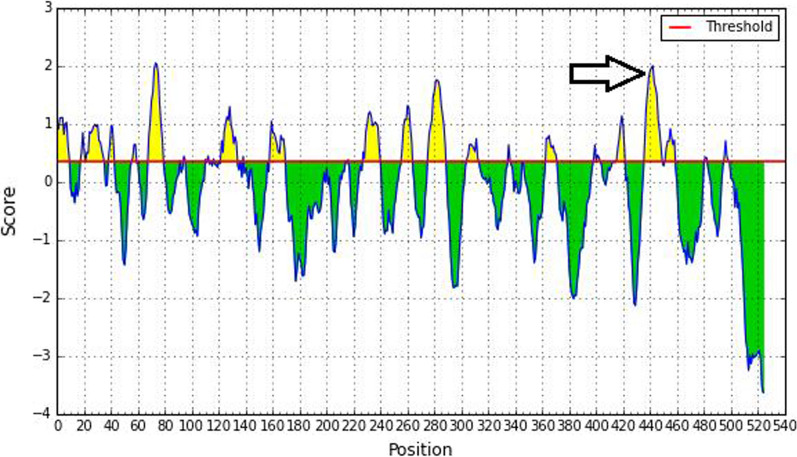
Prediction of B cell epitopes of transmembrane α-CA (W7JAI7) from *P. falciparum*. The arrow indicates a region where there is a high score for affinity between “428-LRTIINVSSAVHVGS-442” peptide sequence and B cell epitopes, as well as high affinity to MHC-II HLA-DRB1*01:03

The prediction of B cell epitopes in *Plasmodium* tmCAs revealed that the epitope prediction process utilized by the IEDB Analysis Resource discriminates positive from negative results with a default 0.35 threshold. There was no overlap between the predicted *Plasmodium* tmCAs (W7JAI7, Q8IHW5, and V7PFH4), MHC-I epitopes (Tables 3 and 4), and potential predicted B cell epitopes. However, the MHC-II epitope peptide sequence "428-LRTIINVSSAVHVGS-442" of transmembrane α-CA (W7JAI7) from *P. falciparum* showed a high predicted affinity score (2.0) to HLA-DRB1*01:03 (Fig. 2).

## Discussion

The complex biology and lifecycle of *Plasmodium* includes several features which make it more challenging for vaccine development as compared to bacteria and viruses. This malaria-causing protozoan passes multiple intra- and extracellular as well as sexual and asexual developmental stages, during which several distinct transcriptional profiles occur, ultimately resulting in the expression of various unique sets of proteins. These molecules might be antigenic or non-antigenic targets for the human immune system at different stages of malaria infection. Therefore, it is not surprising that no efficient malaria vaccine has successfully passed clinical trial [4], except Mosquirix or RTS,S/AS01, which showed some immune response in a specific endemic geographic area with a narrow age limit (children) [39, 41]. A recombinant purine salvage enzyme, hypoxanthine guanine xanthine phosphoribosyl transferase (HGXPRT), from *P. falciparum* has been evaluated as a target antigen [89]. A vaccine targeting this protein was able to immunize mice against *P. yoelii*, although issues arose due to high homology between HGXPRT and the human homolog Hypoxanthine Phosphoribosyltransferase (HGPRT).

Sequence analysis of *Plasmodium* transmembrane α-CAs revealed that three conserved histidines are located in the catalytic active site of α-CAs, while four conserved histidines and one phenylalanine are located in the catalytic active site of η-CAs. Further analysis by MSA showed that *Plasmodium* tmCAs can be categorized in two groups, group 1 (represented by W7JAI7) and group 2 (represented by Q8IHW5). For η-CAs, similarly the V7PFH4 was selected as representative based on the identity results obtained from MSA analysis. Moreover, a separate MSA analysis of human membrane-bound α-CAs (CA4, CA9, CA12, and CA14) and *Plasmodium* transmembrane α-CAs and η-CAs, displayed no significant identity between the two groups. In addition, No significant similarity was detected between the examined *Plasmodium* tmCAs and other hCA crystal structures in PDB database. Therefore, the minimum similarity must be present between tmCAs as the template in vaccine production and human transmembrane α-CAs [90]. As the result, *Plasmodium* transmembrane α- and η-CAs could be proposed as potential specific vaccine targets for malaria prevention without any adverse immunity response towards human membrane-bound α-CAs.

The prediction of subcellular localization of *Plasmodium* α- and η-CAs revealed that each of the three tested tmCAs (W7JAI7, Q8IHW5, and V7PFH4) contains a transmembrane helix near the C-terminal end. A major portion of these proteins, starting from the N-terminus, is predicted to be extracellular and thus accessible to antibody-mediated immunological detection, whereas the residual 1–3 amino acids at the C-terminus are intracellular and not readily accessible.

Predictions based on several bioinformatics tools identified two extracellular peptide sequences in *P. falciparum* α-CAs (group 1 and 2) and one in *P. yoelii* η-CAs, which showed high affinity, stability, and antigenicity. These predictions also had significant scores for epitope binding to human MHC-II: including MHC-II subtype DRB1 for group 1 and 2 of *P. falciparum* α-CAs, and MHC-II subtype DQ for *P. yoelii* η-CAs. Molecular accessibility study of the predicted MHC-ligands of *Plasmodium* tmCAs suggested that they are on the surface and accessible. Therefore, it is conceivable that these tmCAs epitopes can be exposed to the immune system via MHC-I and -II and would be accessible to antibodies.

The allergenicity prediction results of *Plasmodium* tmCAs revealed that the peptides from the group 1 (W7JAI7) and group 2 (Q8IHW5) α-CAs, or *P. yoelii* η-CAs (V7PFH4) are unlikely to cause hypersensitivity if incorporated in vaccines. No cross-reaction was predicted with known allergens, because cross-reactivity rarely occurs when the sequence identity is less than 50%. Most cross-reactions and consequent hypersensitivity take place when there is ≥ 70% identity [81, 91].

Intramolecular disulfide-bonds between Cys residues play a major role in the stability and immunogenicity of antigens and consequently raise the production of neutralizing antibody in the human body, as described for human immunodeficiency virus type 1 (HIV-1) [92], hepatitis B vaccine antigen [93], haemorrhagic septicemia virus (a fish Rhabdovirus) [94], murine leukemia virus (MuLV) [95], and Ebola virus [96]. Another study on surface proteins of merozoites in malaria infection revealed that the recognition of antigen epitopes by antibodies is a disulfide bond-dependent process [97]. Hence, based on disulfide-bond predictions for *Plasmodium* tmCAs, all three tmCAs (W7JAI7, Q8IHW5, and V7PFH4) potentially have enough stability and proper structural folding to be detected by neutralizing antibodies.

The prediction of B cell epitopes in *Plasmodium* tmCAs suggest they can be presented by APCs to B cells to generate a response in the humoral immune system. Therefore, it was concluded that the most suitable target peptide for vaccine development would be “428-LRTIINVSSAVHVGS-442”, which is part of *P. falciparum* transmembrane α-CA (W7JAI7). Evaluation of human HLA alleles, including HLA-DRB1*01:03 from MHC-II, predicted a high affinity for this polypeptide.

This study proposes synthetic peptide vaccines for malaria prevention. Synthetic peptide vaccine technology is one of the safest approaches for vaccine development because it eliminates the risk of malaria transmission, compared with whole protozoa attenuation. Immunization could be enhanced using already established conjugation methods: combining circumsporozoite (CS) protein from *P. falciparum* and a viral carrier, made from hepatitis B surface antigen (HBsAg), has been found to induce immunity and production of anti-CS antibody in mice [98]. In addition, several other new avenues are available for malaria prevention, such as: combination therapies using microRNAs (miRNAs) [99], and virus-like particle based vaccines containing immunogenic peptide sequence from tmCAs or η-CAs.

## Conclusions

The study defined that these highly stable and antigenic epitopes including two extracellular peptide sequences in *P. falciparum* α-CAs (group 1: W7JAI7 and group 2: Q8IHW5) and one in *P. yoelii* η-CAs (V7PFH4) are surface accessible and would be exposed to human immune system and therefore represent potential targets for vaccination against malaria. In addition, it is a necessity to follow the results of present study by further in vitro and in vivo methods to evaluate the potentials of tmCAs as the vaccine candidates against malaria.

## Supplementary Information

**Additional file 1: Figure S1.** W7JAI7 is the representative of group 1 of α-CAs. **Figure S2. **Q8IHW5 is the representative of group 2 of α-CAs. **Figure S3.** V7PFH4 is the representative of η-CAs. **Figure S4. **Multiple sequence analysis (MSA) of W7JAI7 selected from group 1 of transmembrane α-CAs of *Plasmodium* spp. with human transmembrane α-CAs. **Figure S5. **Multiple sequence analysis (MSA) of Q8IHW5 selected from group 2 of transmembrane α-CAs of *Plasmodium* spp. with human transmembrane α-CAs. **Figure S6. **Multiple sequence analysis (MSA) containing transmembrane η-CA (V7PFH4) of *Plasmodium* spp. and human transmembrane α-CAs. **Figure S7.** Prediction of transmembrane localization of representative α- and η-CAs from *Plasmodium *spp. The prediction identified transmembrane segments near the C-terminal ends of W7JAI7 from group 1 (A) and Q8IHW5 from group 2 (B) of *P. falciparum* α-CAs and V7PFH4 from *P. yoelii* η-CAs (C). **Figure S8.** Pairwise sequence alignment of PDB 2W2J and W7JAI7 V7PFH4 indicating the locations of predicted MHC-I ligands. **Figure S9.** Pairwise sequence alignment of PDB 3FE4 and Q8IHW5 indicating the locations of predicted MHC-I ligands. **Figure S10.** Pairwise sequence alignment of PDB 3FE4 and V7PFH4 indicating the locations of predicted MHC-I ligands. **Figure S11.** Pairwise sequence alignment of PDB 2W2J and **W7JAI7 **indicating the locations of predicted MHC-II ligands. **Figure S12.** Pairwise sequence alignment of PDB PDB 3FE4 and Q8IHW5 indicating the location of predicted MHC-II ligand. **Figure S13.** Pairwise sequence alignment of PDB 3FE4 and V7PFH4 indicating the location of predicted MHC-II ligand.

**Additional file 2: Table S1.** Disulfide-bond prediction of *Plasmodium* tmCAs.

## Data Availability

All data in this study have been presented in tables, figures, and Additional files.
