# Supplementary material for: A reverse vaccinology approach on transmembrane carbonic anhydrases from Plasmodium species as vaccine candidates for malaria prevention
Source: Malar J. 2022 Jun 15;21:189. doi: 10.1186/s12936-022-04186-7 (PMC9199335; doi:10.1186/s12936-022-04186-7)
Supplement: Supplementary file 1 — Additional file 1: Figure S1. W7JAI7 is the representative of group 1 of α-CAs. Figure S2. Q8IHW5 is the representative of group 2 of α-CAs. Figure S3. V7PFH4 is the representative of η-CAs. Figure S4. Multiple sequence analysis (MSA) of W7JAI7 selected from group 1 of transmembrane α-CAs of Plasmodium spp. with human transmembrane α-CAs. Figure S5. Multiple sequence analysis (MSA) of Q8IHW5 selected from group 2 of transmembrane α-CAs of Plasmodium spp. with human transmembrane α-CAs. Figure S6. Multiple sequence analysis (MSA) containing transmembrane η-CA (V7PFH4) of Plasmodium spp. and human transmembrane α-CAs. Figure S7. Prediction of transmembrane localization of representative α- and η-CAs from Plasmodium spp. The prediction identified transmembrane segments near the C-terminal ends of W7JAI7 from group 1 (A) and Q8IHW5 from group 2 (B) of P. falciparum α-CAs and V7PFH4 from P. yoelii η-CAs (C). Figure S8. Pairwise sequence alignment of PDB 2W2J and W7JAI7 V7PFH4 indicating the locations of predicted MHC-I ligands. Figure S9. Pairwise sequence alignment of PDB 3FE4 and Q8IHW5 indicating the locations of predicted MHC-I ligands. Figure S10. Pairwise sequence alignment of PDB 3FE4 and V7PFH4 indicating the locations of predicted MHC-I ligands. Figure S11. Pairwise sequence alignment of PDB 2W2J and W7JAI7 indicating the locations of predicted MHC-II ligands. Figure S12. Pairwise sequence alignment of PDB PDB 3FE4 and Q8IHW5 indicating the location of predicted MHC-II ligand. Figure S13. Pairwise sequence alignment of PDB 3FE4 and V7PFH4 indicating the location of predicted MHC-II ligand. [file 12936_2022_4186_MOESM1_ESM.doc]

**tr|W7JAI7|W7JAI7_PLAFA MQKKDEKNIKDFHINDYEIDGKTIHNKENKDSFKMNKNKLNDNEELFYMDNILSYKPNKK**

**tr|A0A024W467|A0A024W467_PLAFA MQKKDEKNIKDFHINDYEIDGKTIHNKENKDSFKMNKNKLNDNEELFYMDNILSYKPNKK**

**tr|A0A024X6P3|A0A024X6P3_PLAFC MQKKDEKNIKDFHINDYEIDGKTIHNKENKDSFKMNKNKLNDNEELFYMDNILSYKPNKK**

**tr|W4IEQ5|W4IEQ5_PLAFA MQKKDEKNIKDFHINDYEIDGKTIHNKENKDSFKMNKNKLNDNEELFYMDNILSYKPNKK**

**tr|W7K3B0|W7K3B0_PLAFO MQKKDEKNIKDFHINDYEIDGKTIHNKENKDSFKMNKNKLNDNEELFYMDNILSYKPNKK**

****************************************************************

**tr|W7JAI7|W7JAI7_PLAFA KLFTYSFSENEGNSEKEETLYNFKNMKNINSVQNNINKTFLYNKLKNVDYYEHGYNWDIG**

**tr|A0A024W467|A0A024W467_PLAFA KLFTYSFSENEGNSEKEETLYNFKNMKNINSVQNNINKTFLYNKLKNVDYYEHGYNWDIG**

**tr|A0A024X6P3|A0A024X6P3_PLAFC KLFTYSFSENEGNSEKEETLYNFKNMKNINSVQNNINKTFLYNKLKNVDYYEHGYNWDIG**

**tr|W4IEQ5|W4IEQ5_PLAFA KLFTYSFSENEGNSEKEETLYNFKNMKNINSVQNNINKTFLYNKLKNVDYYEHGYNWDIG**

**tr|W7K3B0|W7K3B0_PLAFO KLFTYSFSENEGNSEKEETLYNFKNMKNINSVQNNINKTFLYNKLKNVDYYEHGYNWDIG**

****************************************************************

**tr|W7JAI7|W7JAI7_PLAFA QCKTGKYQSPVDLPMKDLKERELKNISDVYLNLFDDDNYAWNNYNKPWMKGDFFYYYEYF**

**tr|A0A024W467|A0A024W467_PLAFA QCKTGKYQSPVDLPMKDLKERELKNISDVYLNLFDDDNYAWNNYNKPWMKGDFFYYYEYF**

**tr|A0A024X6P3|A0A024X6P3_PLAFC QCKTGKYQSPVDLPMKDLKERELKNISDVYLNLFDDDNYAWNNYNKPWMKGDFFYYYEYF**

**tr|W4IEQ5|W4IEQ5_PLAFA QCKTGKYQSPVDLPMKDLKERELKNISDVYLNLFDDDNYAWNNYNKPWMKGDFFYYYEYF**

**tr|W7K3B0|W7K3B0_PLAFO QCKTGKYQSPVDLPMKDLKERELKNISDVYLNLFDDDNYAWNNYNKPWMKGDFFYYYEYF**

****************************************************************

**tr|W7JAI7|W7JAI7_PLAFA IKKIVINRQNNIFQIKAARDGIIPFGVLFTTEQPAMFYADQIHFHAPSEHTFQGSGNRRE**

**tr|A0A024W467|A0A024W467_PLAFA IKKIVINRQNNIFQIKAARDGIIPFGVLFTTEQPAMFYADQIHFHAPSEHTFQGSGNRRE**

**tr|A0A024X6P3|A0A024X6P3_PLAFC IKKIVINRQNNIFQIKAARDGIIPFGVLFTTEQPAMFYADQIHFHAPSEHTFQGSGNRRE**

**tr|W4IEQ5|W4IEQ5_PLAFA IKKIVINRQNNIFQIKAARDGIIPFGVLFTTEQPAMFYADQIHFHAPSEHTFQGSGNRRE**

**tr|W7K3B0|W7K3B0_PLAFO IKKIVINRQNNIFQIKAARDGIIPFGVLFTTEQPAMFYADQIHFHAPSEHTFQGSGNRRE**

****************************************************************

HXH

**tr|W7JAI7|W7JAI7_PLAFA IEMQIFHSTNYFYDIQDDKSKYKKKYGLHIYNNLKKNSKETSKKDSSRYHSYLMSFLMNS**

**tr|A0A024W467|A0A024W467_PLAFA IEMQIFHSTNYFYDIQDDKSKYKKKYGLHIYNNLKKNSKETSKKDSSRYHSYLMSFLMNS**

**tr|A0A024X6P3|A0A024X6P3_PLAFC IEMQIFHSTNYFYDIQDDKSKYKKKYGLHIYNNLKKNSKETSKKDSSRYHSYLMSFLMNS**

**tr|W4IEQ5|W4IEQ5_PLAFA IEMQIFHSTNYFYDIQDDKSKYKKKYGLHIYNNLKKNSKETSKKDSSRYHSYLMSFLMNS**

**tr|W7K3B0|W7K3B0_PLAFO IEMQIFHSTNYFYDIQDDKSKYKKKYGLHIYNNLKKNSKETSKKDSSRYHSYLMSFLMNS**

****************************************************************

H

**tr|W7JAI7|W7JAI7_PLAFA LSNEQLQNKYNKKKRIKKMKNQYEVISITFTSAEINASTINAFKKLPSEKFLRTIINVSS**

**tr|A0A024W467|A0A024W467_PLAFA LSNEQLQNKYNKKKRIKKMKNQYEVISITFTSAEINASTINAFKKLPSEKFLRTIINVSS**

**tr|A0A024X6P3|A0A024X6P3_PLAFC LSNEQLQNKYNKKKRIKKMKNQYEVISITFTSAEINASTINAFKKLPSEKFLRTIINVSS**

**tr|W4IEQ5|W4IEQ5_PLAFA LSNEQLQNKYNKKKRIKKMKNQYEVISITFTSAEINASTINAFKKLPSEKFLRTIINVSS**

**tr|W7K3B0|W7K3B0_PLAFO LSNEQLQNKYNKKKRIKKMKNQYEVISITFTSAEINASTINAFKKLPSEKFLRTIINVSS**

****************************************************************

**tr|W7JAI7|W7JAI7_PLAFA AVHVGSDPTLVELKDALNLDALMMMLNIEDMQFLSYQGSSTLPLCDENVSWKVAKQPLPV**

**tr|A0A024W467|A0A024W467_PLAFA AVHVGSDPTLVELKDALNLDALMMMLNIEDMQFLSYQGSSTLPLCDENVSWKVAKQPLPV**

**tr|A0A024X6P3|A0A024X6P3_PLAFC AVHVGSDPTLVELKDALNLDALMMMLNIEDMQFLSYQGSSTLPLCDENVSWKVAKQPLPV**

**tr|W4IEQ5|W4IEQ5_PLAFA AVHVGSDPTLVELKDALNLDALMMMLNIEDMQFLSYQGSSTLPLCDENVSWKVAKQPLPV**

**tr|W7K3B0|W7K3B0_PLAFO AVHVGSDPTLVELKDALNLDALMMMLNIEDMQFLSYQGSSTLPLCDENVSWKVAKQPLPV**

****************************************************************

**tr|W7JAI7|W7JAI7_PLAFA STETILNFYYLLKKHTPNYSGSDNDNYRSLQNVEDNTRHYRKFSLVQVFPIQVLISSAIS**

**tr|A0A024W467|A0A024W467_PLAFA STETILNFYYLLKKHTPNYSGSDNDNYRSLQNVEDNTRHYRKFSLVQVFPIQVLISSAIS**

**tr|A0A024X6P3|A0A024X6P3_PLAFC STETILNFYYLLKKHTPNYSGSDNDNYRSLQNVEDNTRHYRKFSLVQVFPIQVLISSAIS**

**tr|W4IEQ5|W4IEQ5_PLAFA STETILNFYYLLKKHTPNYSGSDNDNYRSLQNVEDNTRHYRKFSLVQVFPIQVLISSAIS**

**tr|W7K3B0|W7K3B0_PLAFO STETILNFYYLLKKHTPNYSGSDNDNYRSLQNVEDNTRHYRKFSLVQVFPIQVLISSAIS**

****************************************************************

**tr|W7JAI7|W7JAI7_PLAFA NIEDKKVINIIKDISPKNMSFSYYSKWDIYFILFIFYNIVLFLF**

**tr|A0A024W467|A0A024W467_PLAFA NIEDKKVINIIKDISPKNMSFSYYSKWDIYFILFIFYNIVLFLF**

**tr|A0A024X6P3|A0A024X6P3_PLAFC NIEDKKVINIIKDISPKNMSFSYYSKWDIYFILFIFYNIVLFLF**

**tr|W4IEQ5|W4IEQ5_PLAFA NIEDKKVINIIKDISPKNMSFSYYSKWDIYFILFIFYNIVLFLF**

**tr|W7K3B0|W7K3B0_PLAFO NIEDKKVINIIKDISPKNMSFTYYSKWDIYFILFIFYNIVLFLF**

***********************:************************

**Fig. S1** W7JAI7 is the representative of group 1 of α-CAs.

**tr|A0A060RUT7|A0A060RUT7_PLARE MKLLYLLYPILLFYNVNVFINYKKSRLMLEMIEKYNTHFVQTTKPYYEFNVTNLSNSKKK**

**tr|W4J022|W4J022_PLAFP MKLLYLLYPILLFYNVNVFINYKKSRLMLEMIDKYNTHFVQTTKPYYEFNVTNLTNSKKK**

**tr|A0A0L1I6C6|A0A0L1I6C6_PLAFA MKLLYLLYPILLFYSVNVFINYKKSRLMLEMIDKYNTHFVQTTKPYYEFNVTNLTNSKKK**

**tr|Q8IHW5|Q8IHW5_PLAF7 MKLLYLLYPILLFYNVNVFINYKKSRLMLEMIDKYNTHFVQTTKPYYEFNVTNLTNSKKK**

**tr|W7FAY3|W7FAY3_PLAF8 MKLLYLLYPILLFYNVNVFINYKKSRLMLEMIDKYNTHFVQTTKPYYEFNVTNLTNSKKK**

**tr|W7FN76|W7FN76_PLAFA MKLLYLLYPILLFYNVNVFINYKKSRLMLEMIDKYNTHFVQTTKPYYEFNVTNLTNSKKK**

**tr|A0A024WMV1|A0A024WMV1_PLAFA MKLLYLLYPILLFYNVNVFINYKKSRLMLEMIDKYNTHFVQTTKPYYEFNVTNLTNSKKK**

**tr|A0A024UXI3|A0A024UXI3_PLAFA MKLLYLLYPILLFYNVNVFINYKKSRLMLEMIDKYNTHFVQTTKPYYEFNVTNLTNSKKK**

****************.*****************:*********************:*******

**tr|A0A060RUT7|A0A060RUT7_PLARE KKKKKRGNHLIGSGENMQKKDEKHIKDFHINDYEIVGKTIHNKENKDAFKMNKNKLNDNE**

**tr|W4J022|W4J022_PLAFP KKKKERENHLIGSGENMQKKDEKNIKDFHINDYEIDGKTIHNKENKDSFKMNKNKLNDNE**

**tr|A0A0L1I6C6|A0A0L1I6C6_PLAFA KKKKKRENHLIGSGENMQKKDEKNIKDFHINDYEIDGKTIHNKENKDSFKMNKNKLNDNE**

**tr|Q8IHW5|Q8IHW5_PLAF7 KKKKKRENHLIGSGENMQKKDEKNIKDFHINDYEIDGKTIHNKENKDSFKMNKNKLNDNE**

**tr|W7FAY3|W7FAY3_PLAF8 KKKKKRENHLIGSGENMQKKDEKNIKDFHINDYEIDGKTIHNKENKDSFKMNKNKLNDNE**

**tr|W7FN76|W7FN76_PLAFA KKKKKRENHLIGSGENMQKKDEKNIKDFHINDYEIDGKTIHNKENKDSFKMNKNKLNDNE**

**tr|A0A024WMV1|A0A024WMV1_PLAFA KKKKKRENHLIGSGENMQKKDEKNIKDFHINDYEIDGKTIHNKENKDSFKMNKNKLNDNE**

**tr|A0A024UXI3|A0A024UXI3_PLAFA KKKKKRENHLIGSGENMQKKDEKNIKDFHINDYEIDGKTIHNKENKDSFKMNKNKLNDNE**

******:* ****************.*********** ***********:**************

**tr|A0A060RUT7|A0A060RUT7_PLARE ELFYMDSILSYKPNKKKLFTYSFSENEGNSEKEETLYNFENRKNINSVQNNINKTFLYNK**

**tr|W4J022|W4J022_PLAFP ELFYMDNILSYKPNKKKLFTYSFSENEGNSEKEETLYNFKNMKNINSVQNNINKTFLYNK**

**tr|A0A0L1I6C6|A0A0L1I6C6_PLAFA ELFYMDNILSYKPNKKKLFTYSFSENEGNSEKEETLYNFKNMKNINSVQNNINKTFLYNK**

**tr|Q8IHW5|Q8IHW5_PLAF7 ELFYMDNILSYKPNKKKLFTYSFSENEGNSEKEETLYNFKNMKNINSVQNNINKTFLYNK**

**tr|W7FAY3|W7FAY3_PLAF8 ELFYMDNILSYKPNKKKLFTYSFSENEGNSEKEETLYNFKNMKNINSVQNNINKTFLYNK**

**tr|W7FN76|W7FN76_PLAFA ELFYMDNILSYKPNKKKLFTYSFSENEGNSEKEETLYNFKNMKNINSVQNNINKTFLYNK**

**tr|A0A024WMV1|A0A024WMV1_PLAFA ELFYMDNILSYKPNKKKLFTYSFSENEGNSEKEETLYNFKNMKNINSVQNNINKTFLYNK**

**tr|A0A024UXI3|A0A024UXI3_PLAFA ELFYMDNILSYKPNKKKLFTYSFSENEGNSEKEETLYNFKNMKNINSVQNNINKTFLYNK**

********.********************************:* ********************

**tr|A0A060RUT7|A0A060RUT7_PLARE LKNVDYYEHGYNWDIGQCKTGKYQSPVDLPMKDLKERELKNISDVYLNLFDDDNYAWNNY**

**tr|W4J022|W4J022_PLAFP LKNVDYYEHGYNWDIGQCKTGKYQSPVDLPMKDLKERELKNISDVYLNLFDDDNYAWNNY**

**tr|A0A0L1I6C6|A0A0L1I6C6_PLAFA LKNVDYYEHGYNWDIGQCKTGKYQSPVDLPMKDLKERELKNISDVYLNLFDDDNYAWNNY**

**tr|Q8IHW5|Q8IHW5_PLAF7 LKNVDYYEHGYNWDIGQCKTGKYQSPVDLPMKDLKERELKNISDVYLNLFDDDNYAWNNY**

**tr|W7FAY3|W7FAY3_PLAF8 LKNVDYYEHGYNWDIGQCKTGKYQSPVDLPMKDLKERELKNISDVYLNLFDDDNYAWNNY**

**tr|W7FN76|W7FN76_PLAFA LKNVDYYEHGYNWDIGQCKTGKYQSPVDLPMKDLKERELKNISDVYLNLFDDDNYAWNNY**

**tr|A0A024WMV1|A0A024WMV1_PLAFA LKNVDYYEHGYNWDIGQCKTGKYQSPVDLPMKDLKERELKNISDVYLNLFDDDNYAWNNY**

**tr|A0A024UXI3|A0A024UXI3_PLAFA LKNVDYYEHGYNWDIGQCKTGKYQSPVDLPMKDLKERELKNISDVYLNLFDDDNYAWNNY**

****************************************************************

**tr|A0A060RUT7|A0A060RUT7_PLARE NKPWMKGDFFYYYECFIKKIVINRQNNIFQIKAARDGIIPFGVLFTTEEPAMFYADQIHF**

**tr|W4J022|W4J022_PLAFP NKPWMKGDFFYYYEYFIKKIVINRQNNIFQIKAARDGIIPFGVLFTTEQPAMFYADQIHF**

**tr|A0A0L1I6C6|A0A0L1I6C6_PLAFA NKPWMKGDFFYYYEYFIKKIVINRQNNIFQIKAARDGIIPFGVLFTTEQPAMFYADQIHF**

**tr|Q8IHW5|Q8IHW5_PLAF7 NKPWMKGDFFYYYEYFIKKIVINRQNNIFQIKAARDGIIPFGVLFTTEQPAMFYADQIHF**

**tr|W7FAY3|W7FAY3_PLAF8 NKPWMKGDFFYYYEYFIKKIVINRQNNIFQIKAARDGIIPFGVLFTTEQPAMFYADQIHF**

**tr|W7FN76|W7FN76_PLAFA NKPWMKGDFFYYYEYFIKKIVINRQNNIFQIKAARDGIIPFGVLFTTEQPAMFYADQIHF**

**tr|A0A024WMV1|A0A024WMV1_PLAFA NKPWMKGDFFYYYEYFIKKIVINRQNNIFQIKAARDGIIPFGVLFTTEQPAMFYADQIHF**

**tr|A0A024UXI3|A0A024UXI3_PLAFA NKPWMKGDFFYYYEYFIKKIVINRQNNIFQIKAARDGIIPFGVLFTTEQPAMFYADQIHF**

**************** *********************************:*************

HXH

**tr|A0A060RUT7|A0A060RUT7_PLARE HAPSEHTFQGSGNRREIEMQIFHSTNYFYDIQDDKSKYKKKYGLHIYNNLKKNSKETSKK**

**tr|W4J022|W4J022_PLAFP HAPSEHTFQGSGNRREIEMQIFHSTNYFYDIQDDKSKYKKKYGLHIYNNLKKNSKETSKK**

**tr|A0A0L1I6C6|A0A0L1I6C6_PLAFA HAPSEHTFQGSGNRREIEMQIFHSTNYFYDIQDDKSKYKKKYGLHIYNNLKKNSKETSKK**

**tr|Q8IHW5|Q8IHW5_PLAF7 HAPSEHTFQGSGNRREIEMQIFHSTNYFYDIQDDKSKYKKKYGLHIYNNLKKNSKETSKK**

**tr|W7FAY3|W7FAY3_PLAF8 HAPSEHTFQGSGNRREIEMQIFHSTNYFYDIQDDKSKYKKKYGLHIYNNLKKNSKETSKK**

**tr|W7FN76|W7FN76_PLAFA HAPSEHTFQGSGNRREIEMQIFHSTNYFYDIQDDKSKYKKKYGLHIYNNLKKNSKETSKK**

**tr|A0A024WMV1|A0A024WMV1_PLAFA HAPSEHTFQGSGNRREIEMQIFHSTNYFYDIQDDKSKYKKKYGLHIYNNLKKNSKETSKK**

**tr|A0A024UXI3|A0A024UXI3_PLAFA HAPSEHTFQGSGNRREIEMQIFHSTNYFYDIQDDKSKYKKKYGLHIYNNLKKNSKETSKK**

****************************************************************

H

**tr|A0A060RUT7|A0A060RUT7_PLARE DSSRYHSYLMSYLMNSLSNEQLQNKYNKKKRIKKMKNQYEVISITFTSAEINASTINAFK**

**tr|W4J022|W4J022_PLAFP DSSRYHSYLMSFLMNSLSNEQLQNKYNKKKRIKKMKNQYEVISITFTSAEINASTINAFK**

**tr|A0A0L1I6C6|A0A0L1I6C6_PLAFA DSSRYHSYLMSFLMNSLSNEQLQNKYNKKKRIKKMKNQYEVISITFTSAEINASTINAFK**

**tr|Q8IHW5|Q8IHW5_PLAF7 DSSRYHSYLMSFLMNSLSNEQLQNKYNKKKRIKKMKNQYEVISITFTSAEINASTINAFK**

**tr|W7FAY3|W7FAY3_PLAF8 DSSRYHSYLMSFLMNSLSNEQLQNKYNKKKRIKKMKNQYEVISITFTSAEINASTINAFK**

**tr|W7FN76|W7FN76_PLAFA DSSRYHSYLMSFLMNSLSNEQLQNKYNKKKRIKKMKNQYEVISITFTSAEINASTINAFK**

**tr|A0A024WMV1|A0A024WMV1_PLAFA DSSRYHSYLMSFLMNSLSNEQLQNKYNKKKRIKKMKNQYEVISITFTSAEINASTINAFK**

**tr|A0A024UXI3|A0A024UXI3_PLAFA DSSRYHSYLMSFLMNSLSNEQLQNKYNKKKRIKKMKNQYEVISITFTSAEINASTINAFK**

*************:**************************************************

**tr|A0A060RUT7|A0A060RUT7_PLARE KLPSEKFLRTIINVSSAVHVGSDPTLVELKEALNLDALMMMLNIEDMQFLSYQGSSTLPL**

**tr|W4J022|W4J022_PLAFP KLPSEKFLRTIINVSSAVHVGSDPTLVELKDALNLDALMMMLNIEDMQFLSYQGSSTLPL**

**tr|A0A0L1I6C6|A0A0L1I6C6_PLAFA KLPSEKFLRTIINVSSAVHVGSDPTLVELKDALNLDALMMMLNIEDMQFLSYQGSSTLPL**

**tr|Q8IHW5|Q8IHW5_PLAF7 KLPSEKFLRTIINVSSAVHVGSDPTLVELKDALNLDALMMMLNIEDMQFLSYQGSSTLPL**

**tr|W7FAY3|W7FAY3_PLAF8 KLPSEKFLRTIINVSSAVHVGSDPTLVELKDALNLDALMMMLNIEDMQFLSYQGSSTLPL**

**tr|W7FN76|W7FN76_PLAFA KLPSEKFLRTIINVSSAVHVGSDPTLVELKDALNLDALMMMLNIEDMQFLSYQGSSTLPL**

**tr|A0A024WMV1|A0A024WMV1_PLAFA KLPSEKFLRTIINVSSAVHVGSDPTLVELKDALNLDALMMMLNIEDMQFLSYQGSSTLPL**

**tr|A0A024UXI3|A0A024UXI3_PLAFA KLPSEKFLRTIINVSSAVHVGSDPTLVELKDALNLDALMMMLNIEDMQFLSYQGSSTLPL**

********************************:*******************************

**tr|A0A060RUT7|A0A060RUT7_PLARE CDENVSWKVAKQPLPVSTETILNFYYLLKKHTPNYSGSDNDNYRSLQNVEDNTRHYRKFS**

**tr|W4J022|W4J022_PLAFP CDENVSWKVAKQPLPVSTETILNFYYLLKKHTPNYSGSDNDNYRSLQNVEDNTRHYRKFS**

**tr|A0A0L1I6C6|A0A0L1I6C6_PLAFA CDENVSWKVAKQPLPVSTETILNFYYLLKKHTPNYSGSDNDNYRSLQNVEDNTRHYRKFS**

**tr|Q8IHW5|Q8IHW5_PLAF7 CDENVSWKVAKQPLPVSTETILNFYYLLKKHTPNYSGSDNDNYRSLQNVEDNTRHYRKFS**

**tr|W7FAY3|W7FAY3_PLAF8 CDENVSWKVAKQPLPVSTETILNFYYLLKKHTPNYSGSDNDNYRSLQNVEDNTRHYRKFS**

**tr|W7FN76|W7FN76_PLAFA CDENVSWKVAKQPLPVSTETILNFYYLLKKHTPNYSGSDNDNYRSLQNVEDNTRHYRKFS**

**tr|A0A024WMV1|A0A024WMV1_PLAFA CDENVSWKVAKQPLPVSTETILNFYYLLKKHTPNYSGSDNDNYRSLQNVEDNTRHYRKFS**

**tr|A0A024UXI3|A0A024UXI3_PLAFA CDENVSWKVAKQPLPVSTETILNFYYLLKKHTPNYSGSDNDNYRSLQNVEDNTRHYRKFS**

****************************************************************

**tr|A0A060RUT7|A0A060RUT7_PLARE LVQVFPIQVLISSAISNVEDKEVINIIKDISPKNMSFSYYSKWDIYFILFIFYNIVLFLF**

**tr|W4J022|W4J022_PLAFP LVQVFPIQVLISSAISNIEDKKVINIIKDISPKNMSFSYYSKWDIYFILFIFYNIVLFLF**

**tr|A0A0L1I6C6|A0A0L1I6C6_PLAFA LVQVFPIQVLISSAISNIEDKKVINIIKDISPKNMSFSYYSKWDIYFILFIFYNIVLFLF**

**tr|Q8IHW5|Q8IHW5_PLAF7 LVQVFPIQVLISSAISNIEDKKVINIIKDISPKNMSFTYYSKWDIYFILFIFYNIVLFLF**

**tr|W7FAY3|W7FAY3_PLAF8 LVQVFPIQVLISSAISNIEDKKVINIIKDISPKNMSFSYYSKWDIYFILFIFYNIVLFLF**

**tr|W7FN76|W7FN76_PLAFA LVQVFPIQVLISSAISNIEDKKVINIIKDISPKNMSFSYYSKWDIYFILFIFYNIVLFLF**

**tr|A0A024WMV1|A0A024WMV1_PLAFA LVQVFPIQVLISSAISNIEDKKVINIIKDISPKNMSFSYYSKWDIYFILFIFYNIVLFLF**

**tr|A0A024UXI3|A0A024UXI3_PLAFA LVQVFPIQVLISSAISNIEDKKVINIIKDISPKNMSFSYYSKWDIYFILFIFYNIVLFLF**

*******************:***:***************:************************

**Fig. S2** Q8IHW5 is the representative of group 2 of α-CAs.

**tr|A0A077TKL1|A0A077TKL1_PLACH MKHIIFLSIVFCFCDHVVSNNYVGRILFELPDNIMDDLSSGPIVEYEIKEHKDDNPDINK**

**tr|W7AWR1|W7AWR1_PLAVN MKHIIFLSIVFCFCDNVVYNNYVARILFELPDNIIYDLNSSPIVEYEIKEHKDDNPDINK**

**tr|A0A077XD25|A0A077XD25_PLABA MKHIIFLSIVLCFCDNVMYNNYVERILFELPNNITDDLNSEPMVEYEVKEKKDDNIDINK**

**tr|V7PFH4|V7PFH4_9APIC MKHIIFLSIVLCFCDNVMYNNYVERMLFELPNNITDDLNSDPIVEYKIKEKKNDNIDINK**

**tr|A0A078KA92|A0A078KA92_9APIC MKHIIFLSIVLCFCDNVMYNNYVERMLFELPNNITDDLNSDPIVEYKIKEKKNDNIDINK**

**tr|Q7RRH6|Q7RRH6_PLAYO MKHIIFLSIVLCFCDNVMYNNYVERMLFELPNNITDDLNSDPIVEYKIKEKKNDNIDINK**

************:****.*: **** *:*****:** **.* *:***::**:*:** ******

**tr|A0A077TKL1|A0A077TKL1_PLACH DARHWNIEINGDKDNPNIQRSNEGND----------------------------------**

**tr|W7AWR1|W7AWR1_PLAVN EVRHWNIEINEHKDNPNIQRSNEGND----------------------------------**

**tr|A0A077XD25|A0A077XD25_PLABA DVRHWNIEINEHKDNPNIQRNPEGNDNNH-------------------------------**

**tr|V7PFH4|V7PFH4_9APIC DVRHWDIEINEHKDDPNIQRNIEWHDNNDGNGNNNDNNNGNNSGNNSGNNNGNNSGNNND**

**tr|A0A078KA92|A0A078KA92_9APIC DVRHWDIEINEHKDDPNIQRNIEWHDNNDGNGN--------NSGNNSGNNNGNNSGNNND**

**tr|Q7RRH6|Q7RRH6_PLAYO DVRHWDIEINEHKDDPNIQRNIEWHDNNDGNGN--------NSGNNSGNNNGNNSGNNND**

**:.***:**** .**:*****. * .***

**tr|A0A077TKL1|A0A077TKL1_PLACH -------DNNDNWQYHSNYNDKQSESQNENERNEFSLKNEMEKKPEEIKDTQFDKYNEYD**

**tr|W7AWR1|W7AWR1_PLAVN -------DSNNNRQYHSNYNDEQSESQNENERNEFSLKNEAEKNTEERKDTQFDKYNEYD**

**tr|A0A077XD25|A0A077XD25_PLABA -----NNENNDNWEYHSNYNDEKFESQNENERNGFSLKNEVEKNPEERKDTPFDEYNEYA**

**tr|V7PFH4|V7PFH4_9APIC DNNDNDYGNDKNWEYNSNYNDEEFERQNENERDEFSLKNEVEKNSEERKERAFDESNEYA**

**tr|A0A078KA92|A0A078KA92_9APIC DNNDNDYGNDKNWEYNSNYNDEEFERQNENERDEFSLKNEVEKNSEERKERAFDESNEYA**

**tr|Q7RRH6|Q7RRH6_PLAYO DNNDNDYGNDKNWEYNSNYNDEEFERQNENERDEFSLKNEVEKNSEERKERAFDESNEYA**

**.:.* :*.*****:: * ******: ****** **: ** *: **: *****

**tr|A0A077TKL1|A0A077TKL1_PLACH DFENVDN-----NFEENKRKHFEDMQSEDMEDKKRADNKEYADWIEDKKRSDNRGYTGGM**

**tr|W7AWR1|W7AWR1_PLAVN NFENMNN-----NFEENKKKHFEAMQSEDMEDKKRMDNKEYVDWIEDKQREDNRDYANGM**

**tr|A0A077XD25|A0A077XD25_PLABA NFENMNN-----NFEKNKKKYFEDMQSTYMEDKKNVDNKEYMDEVKNKKIEYQ-------**

**tr|V7PFH4|V7PFH4_9APIC DFENMNDLENMNNIEKEKKKYFEDMQSKYHQ-----------------------------**

**tr|A0A078KA92|A0A078KA92_9APIC DFENMNDLENMNNIEKEKKKYFEDMQSKYVEDNTSDGNKEYMGEMKNQQNEYEQNEH---**

**tr|Q7RRH6|Q7RRH6_PLAYO DFENMNDLENMNNIEKEKKKYFEDMQSKYVEDNTSDGNKEYMGEMKNQQNEYEQNEH---**

**:***::: *:*::*:*:** *** :**

**tr|A0A077TKL1|A0A077TKL1_PLACH EDKNSAGNRSYIDGVEDKNSASNKEYISWMDDKNSASNKGYAGWTDDK------------**

**tr|W7AWR1|W7AWR1_PLAVN EDKSSAGNREYIDGMEDKKGEENKDYIDGMEDKKGEENKDYIGWVEDKNIAGNREYIGGM**

**tr|A0A077XD25|A0A077XD25_PLABA ------------------------------------------------------------**

**tr|V7PFH4|V7PFH4_9APIC -------------------------------------QNEYEQ-----NEHQQNEYEQ--**

**tr|A0A078KA92|A0A078KA92_9APIC -QKN-----EYEQNEH-----QQNEYEQN--------------------EHQQNEYEQ--**

**tr|Q7RRH6|Q7RRH6_PLAYO -QKN-----EYEQNEH-----QQNEYEQNE-----HQQNEYEQ-----NEHQQNEYEQ--**

**tr|A0A077TKL1|A0A077TKL1_PLACH ---NGASNKEYAGWRDDKQIEDHRNEENNTKSDTTQDNDNLSFDYSKQGVNWDVGVCKNG**

**tr|W7AWR1|W7AWR1_PLAVN EYKKEAENKEYAGWMNDKQIEDHRNEENNIKSDTTQHNDNLSFDYSKQGMDWAAGVCKNG**

**tr|A0A077XD25|A0A077XD25_PLABA -----------------------KNEENNIKNGIIQYNDNLSFDYSKHGMDWNVGICKNG**

**tr|V7PFH4|V7PFH4_9APIC ---NEHQQNEYEQNEHQQYEQNEQNEEGNIKNGMIQNNENLSFNYAKHGMDWNVGICKNG**

**tr|A0A078KA92|A0A078KA92_9APIC ---NEHQQNEYEQNEHQQYEQNEQNEEGNIKNGMIQNNENLSFNYAKHGMDWNVGICKNG**

**tr|Q7RRH6|Q7RRH6_PLAYO ---NEHQQNEYEQNEHQQYEQNEQNEEGNIKNGMIQNNENLSFNYAKHGMDWNVGICKNG**

**:*** * *. * *:****:*:*:*::* .*:******

**tr|A0A077TKL1|A0A077TKL1_PLACH KYQSPVDLHMHTLKERELKNLSDFYLNAFYDNDEYSWNNYNRPWFKGDIFYYYENLINKI**

**tr|W7AWR1|W7AWR1_PLAVN KYQSPVDLHMHTLKERELKNLSDFYLNAFYDNDEYSWNNYNRPWFKGDIFYYYENLINKI**

**tr|A0A077XD25|A0A077XD25_PLABA KYQSPVDLHMHTLKERELKNLSDFYLNAFYDNDEYSWNNYNKPWFKGDIFYYYENLINKI**

**tr|V7PFH4|V7PFH4_9APIC KYQSPVDLHMHTLKERELKNLSDFYLNAFYDNDEYSWNNFNRPWFKGDIFYYYENLINKI**

**tr|A0A078KA92|A0A078KA92_9APIC KYQSPVDLHMHTLKERELKNLSDFYLNAFYDNDEYSWNNFNRPWFKGDIFYYYENLINKI**

**tr|Q7RRH6|Q7RRH6_PLAYO KYQSPVDLHMHTLKERELKNLSDFYLNAFYDNDEYSWNNFNRPWFKGDIFYYYENLINKI**

*****************************************:*:********************

**tr|A0A077TKL1|A0A077TKL1_PLACH IINRQNNMFKIKASNNEIIPFGVLFTTDEPAIFYSHHINFHSPSEHTFEGSGNRRHIEMQ**

**tr|W7AWR1|W7AWR1_PLAVN IINRQNNMFKIKATNNEIIPFGVLFTTDEPAIFYSHHINFHSPSEHTFEGSGNRRHIEMQ**

**tr|A0A077XD25|A0A077XD25_PLABA IINRQNNMFKIKASNNEIIPFGVLFTTDEPAIFYSHHINFHSPSEHTFEGSGNRRHIEMQ**

**tr|V7PFH4|V7PFH4_9APIC IINRQNNMFKIKASNNEIIPFGVLFTTDEPTIFYSHHINFHSPSEHTFEGSGNRRHIEMQ**

**tr|A0A078KA92|A0A078KA92_9APIC IINRQNNMFKIKASNNEIIPFGVLFTTDEPTIFYSHHINFHSPSEHTFEGSGNRRHIEMQ**

**tr|Q7RRH6|Q7RRH6_PLAYO IINRQNNMFKIKASNNEIIPFGVLFTTDEPTIFYSHHINFHSPSEHTFEGSGNRRHIEMQ**

***************:****************:*******************************

HHXXXH

F

**tr|A0A077TKL1|A0A077TKL1_PLACH IYHSTNEIYDYDESKWNGILGKKKNQKKNNETNIQHSYILTFLRNSLSNPHLGHQNTKNK**

**tr|W7AWR1|W7AWR1_PLAVN IYHSTNEIYDYDESKWNGIFGKKKNQKKNNETNIKHSYILTFLRNSLSNPRLGHQNPKNK**

**tr|A0A077XD25|A0A077XD25_PLABA IYHSTNEIYDYDENKWNGVFGKKTYKKKNNETNIQHSYILTFLMNSLSNPHLSQQYTKNK**

**tr|V7PFH4|V7PFH4_9APIC IYHSTNEIYDYDENKWNGVFEKKNYKKKNNETNIQHSYILTFLMNSLSNPHLGQQYTKNK**

**tr|A0A078KA92|A0A078KA92_9APIC IYHSTNEIYDYDENKWNGVFEKKNYKKKNNETNIQHSYILTFLMNSLSNPHLGQQYTKNK**

**tr|Q7RRH6|Q7RRH6_PLAYO IYHSTNEIYDYDENKWNGVFEKKNYKKKNNETNIQHSYILTFLMNSLSNPHLGQQYTKNK**

***************.****:: **. :********:******** ******:*.:* *****

H

**tr|A0A077TKL1|A0A077TKL1_PLACH KRNKRSKS-YNNIQLGRNGKNTKRINQYQVISITFSSAEIDNSTINNFKKLPSEKFLKTI**

**tr|W7AWR1|W7AWR1_PLAVN KRNKRSKS-YNNTQLGRNGKNTKRLNQYQVISITFSSAEINKSTINNFKKLPSEKFLKTI**

**tr|A0A077XD25|A0A077XD25_PLABA KRNKRSKS-YNSIRMGRNDKNTKRESQYQVISITFSSAEIDKSTINNFKKLPSEKFLKTI**

**tr|V7PFH4|V7PFH4_9APIC KRNKRSKSLYNSIRLDENGKNTKRENQYQVISITFSSAEIDKSTINNFKKLPSEKFLKTI**

**tr|A0A078KA92|A0A078KA92_9APIC KRNKRSKSLYNSIRLDENGKNTKRENQYQVISITFSSAEIDKSTINNFKKLPSEKFLKTI**

**tr|Q7RRH6|Q7RRH6_PLAYO KRNKRSKSLYNSIRLDENGKNTKRENQYQVISITFSSAEIDKSTINNFKKLPSEKFLKTI**

********** **. :: .* ***** .**************::********************

**tr|A0A077TKL1|A0A077TKL1_PLACH LEGTQNIPVGSDPTLVDLKVPLNLNSVLMMLNMKSMEFFAYHGSSTTPDCSENVHWKVAK**

**tr|W7AWR1|W7AWR1_PLAVN LEGTQNVPVGSDPTLVDLKAPLNLNSVLMMLNMKSMEFFAYHGSSTSPDCNENVHWKVAK**

**tr|A0A077XD25|A0A077XD25_PLABA LEGSQNVPVGSGPKLVNLKEPLNLNSLLMMLNMKSMEFFAYHGSSTSPGCNENVHWKVAK**

**tr|V7PFH4|V7PFH4_9APIC LEASQNVPVGSDPKLVNLKEPLNLNSLLMMLNMKSMEFFAYHGSSTSPDCNENVHWKVAK**

**tr|A0A078KA92|A0A078KA92_9APIC LEASQNVPVGSDPKLVNLKKPLNLNSLLMMLNMKSMEFFAYHGSSTSPDCNENVHWKVAK**

**tr|Q7RRH6|Q7RRH6_PLAYO LEASQNVPVGSDPKLVNLKKPLNLNSLLMMLNMKSMEFFAYHGSSTSPDCNENVHWKVAK**

****.:**:**** *.**:** ******:*******************:* *.***********

**tr|A0A077TKL1|A0A077TKL1_PLACH KSLPISTETMLKFYNMLKKTTPDYNSSDNDNFRALQNVQGNVHNYGRVYLIQGFPVQLLI**

**tr|W7AWR1|W7AWR1_PLAVN KSLPISTETMLKFYNMLKKTTPDYNGSDNDNFRALQNVQGNIHNYGRVYLIQGFPVQLLI**

**tr|A0A077XD25|A0A077XD25_PLABA KSLPISTETMLKFYNMLKKTTPYYNASDNDNFRALQNVQGNIHNYGRVYLIQGFPVQLLI**

**tr|V7PFH4|V7PFH4_9APIC KSLPISTETMLKFYNMLKKTTPDYNASDNDNFRALQNVQGNIHNYGRVYLIQGFPVQLLI**

**tr|A0A078KA92|A0A078KA92_9APIC KSLPISTETMLKFYNMLKKTTPDYNASDNDNFRALQNVQGNIHNYGRVYLIQGFPVQLLI**

**tr|Q7RRH6|Q7RRH6_PLAYO KSLPISTETMLKFYNMLKKTTPDYNASDNDNFRALQNVQGNIHNYGRVYLIQGFPVQLLI**

************************ **.***************:********************

**tr|A0A077TKL1|A0A077TKL1_PLACH SSILTTSEDKTVIENIKQAYSKSNGNYIYFNFIFLLLIFMYLQNY**

**tr|W7AWR1|W7AWR1_PLAVN SSILTTSEDKTVIENIKQAYSKSNGNYICFNFIFLLLIFMFLQNY**

**tr|A0A077XD25|A0A077XD25_PLABA SSALMTSDDKNVIENIKLAYSKSSANYIYFNFIFLLLIFMFLQNY**

**tr|V7PFH4|V7PFH4_9APIC SSALTTSEDKNVIENIKLAYSKSSGNYIYFNLIFLLLIFIFLQNY**

**tr|A0A078KA92|A0A078KA92_9APIC SSALTTSEDKNVIENIKLAYSKSSGNYIYFNLIFLLLIFMFLQNY**

**tr|Q7RRH6|Q7RRH6_PLAYO SSALTTSEDKNVIENIKLAYSKSSGNYIYFNLIFLLLIFMFLQNY**

**** * **:**.****** *****..*** **:*******::******

**Fig. S3** V7PFH4 is the representative of η-CAs.

**tr|W7JAI7|W7JAI7_PLAFA ------------------------------------------MQKKDEKNIKDFHINDYE**

**sp|P22748|CAH4_HUMAN ------------------------------------------------------------**

**sp|Q16790|CAH9_HUMAN MAPLCPSPWLPLLIPAPAPGLTVQLLLSLLLLVPVHPQRLPRMQEDSPLGGGS-SGEDDP**

**sp|O43570|CAH12_HUMAN ------------------------------------------------------------**

**sp|Q9ULX7|CAH14_HUMAN ------------------------------------------------------------**

**tr|W7JAI7|W7JAI7_PLAFA IDGKTIHNKEN--KDSFKMNKNKLNDNEELFYMDNILSYKPNK-----KKLFTYSFSENE**

**sp|P22748|CAH4_HUMAN -----------------------------------------------MRMLLALLALS--**

**sp|Q16790|CAH9_HUMAN LGEEDLPSEEDSPREEDPPGEEDLPGEEDLPGEEDLPEVKPKSEEEGSLKLEDLPTVEAP**

**sp|O43570|CAH12_HUMAN ---------------------------------------MPRRSLHAAAVLLLVILKEQ-**

**sp|Q9ULX7|CAH14_HUMAN -------------------------------------------------MLFSALLLEV-**

*** .**

**tr|W7JAI7|W7JAI7_PLAFA GNSEKEETLYNFKNMKNINSVQNNINKTFLYNKLKNVDYYEHGYNWDIGQCKTGKYQSPV**

**sp|P22748|CAH4_HUMAN -----------------AARPSASAESHWCYEVQAESSNYPCLVPVKWGGNCQKDRQSPI**

**sp|Q16790|CAH9_HUMAN GDPQEPQN-------NAHRDKEGDDQSHWRYG---GDPPWPRVSP-----ACAGRFQSPV**

**sp|O43570|CAH12_HUMAN -----------------PSSPAPVNGSKWTYFGPDGENSWSKKYP-----SCGGLLQSPI**

**sp|Q9ULX7|CAH14_HUMAN -----------------IWILAADGGQHWTYEGPHGQDHWPASYP-----ECGNNAQSPI**

**. : * : ***:**

**tr|W7JAI7|W7JAI7_PLAFA DLPMKDLKERELKNISDVYLNLFDDDNYAWNNYNKPWMKGDFFYYYEYFIKKIVINRQNN**

**sp|P22748|CAH4_HUMAN NIVTTKAKVDK--K-----LG-----RFFFSGYDKK--------------QTWTV--QNN**

**sp|Q16790|CAH9_HUMAN DIRPQLAAFCP--A-----LR-----PLELLGFQLPPL------------PELRL--RNN**

**sp|O43570|CAH12_HUMAN DLHSDILQYDA--S-----LT-----PLEFQGYNLSAN------------KQFLL--TNN**

**sp|Q9ULX7|CAH14_HUMAN DIQTDSVTFDP--D-----LP-----ALQPHGYDQPGT------------EPLDL--HNN**

**:: * :: : ****

**tr|W7JAI7|W7JAI7_PLAFA IFQIKAARDGIIPFGVLF-TTEQPAMFYADQIHFHAP-------SEHTFQGSGNRREIEM**

**sp|P22748|CAH4_HUMAN GHSVM----MLLENKASISGGGLPAPYQAKQLHLHWSDLPYK-GSEHSLDGE--HFAMEM**

**sp|Q16790|CAH9_HUMAN GHSVQ----LTLPPGLEM-ALGPGREYRALQLHLHWGAAGRP-GSEHTVEGH--RFPAEI**

**sp|O43570|CAH12_HUMAN GHSVK----LNLPSDMHI-Q-GLQSRYSATQLHLHWGNPNDPHGSEHTVSGQ--HFAAEL**

**sp|Q9ULX7|CAH14_HUMAN GHTVQ----LSLPSTLYL-G-GLPRKYVAAQLHLHWGQKGSPGGSEHQINSE--ATFAEL**

**. : : : : * *:*:* *** ... *:**

HXH

**tr|W7JAI7|W7JAI7_PLAFA QIFHSTNYFYDIQDDKSKYKKKYGLHIYNNLKKNSKETSKKDSSRYHSYLMSFLMNSLSN**

**sp|P22748|CAH4_HUMAN HIVHEKEKGTSRNVKE-AQDPEDEIAVL-----------------------AFLVEA---**

**sp|Q16790|CAH9_HUMAN HVVHLST-AFA-RVDE-ALGRPGGLAVL-----------------------AAFLEE---**

**sp|O43570|CAH12_HUMAN HIVHYNSDLYP-DAST-ASNKSEGLAVL-----------------------AVLIEM---**

**sp|Q9ULX7|CAH14_HUMAN HIVHYDSDSYD-SLSE-AAERPQGLAVL-----------------------GILIEV---**

**::.* . : : . :::**

H

**tr|W7JAI7|W7JAI7_PLAFA EQLQNKYNKKKRIKKMKNQYEVISITFTSAEINASTINAFKKLPSEKFLRTIINVSSAVH**

**sp|P22748|CAH4_HUMAN --------------------------------GTQVNEGFQP-----LVEALSN---IPK**

**sp|Q16790|CAH9_HUMAN --------------------------------GPEENSAYEQ-----LLSRLEE---IAE**

**sp|O43570|CAH12_HUMAN --------------------------------GSF-NPSYDK-----IFSHLQH---VKY**

**sp|Q9ULX7|CAH14_HUMAN --------------------------------GETKNIAYEH-----ILSHLHE---VRH**

**.:. :. : .**

**tr|W7JAI7|W7JAI7_PLAFA VGSDPTLVELKDALNLDALMMMLNIEDMQFLSYQGSSTLPLCDENVSWKVAKQPLPVSTE**

**sp|P22748|CAH4_HUMAN PEMSTTM----AESSLLDLLP-KEEKLRHYFRYLGSLTTPTCDEKVVWTVFREPIQLHRE**

**sp|Q16790|CAH9_HUMAN EGSETQV----PGLDISALLP-S--DFSRYFQYEGSLTTPPCAQGVIWTVFNQTVMLSAK**

**sp|O43570|CAH12_HUMAN KGQEAFV----PGFNIEELLP-E--RTAEYYRYRGSLTTPPCNPTVLWTVFRNPVQISQE**

**sp|Q9ULX7|CAH14_HUMAN KDQKTSV----PPFNLRELLP-K--QLGQYFRYNGSLTTPPCYQSVLWTVFYRRSQISME**

**. : .: *: .: * ** * * * * *.* . : :**

**tr|W7JAI7|W7JAI7_PLAFA TILNFYYLLKKHTP--NYSGSDNDNYRSLQNVEDNTRHYRKFSLVQVFPIQVLISSAISN**

**sp|P22748|CAH4_HUMAN QILAFSQKLYYDKE---QTVSMKDNVRPLQQLGQRT----------------VIKSGAPG**

**sp|Q16790|CAH9_HUMAN QLHTLSDTLWGPG-----DSRLQLNFRATQPLNGRV----------------IEASFPAG**

**sp|O43570|CAH12_HUMAN QLLALETALYCTHMDDPSPREMINNFRQVQKFDERL----------------VYTSFSQV**

**sp|Q9ULX7|CAH14_HUMAN QLEKLQGTLFSTEE--EPSKLLVQNYRALQPLNQRM----------------VFASFIQA**

**: : * * * * . . : ***

**tr|W7JAI7|W7JAI7_PLAFA IEDKKVINIIKDISPKNMSFSYYSKWDIY-------FI-------LFIFYNIVLFLF---**

**sp|P22748|CAH4_HUMAN R-------------PL--------PWALPALLGPMLACLLAGFLR---------------**

**sp|Q16790|CAH9_HUMAN VDSSP-----RAAEPVQL----NSCLAAG----DILALVF-----GLLFAVTSVAFLVQM**

**sp|O43570|CAH12_HUMAN ----------------------QVCTAAGLSLGIILSLALAGILGICIVVVVSIWLFRRK**

**sp|Q9ULX7|CAH14_HUMAN ----------------------GSSYTTG----EMLSLGV-GILVGCLCLLLAVYFIARK**

**tr|W7JAI7|W7JAI7_PLAFA --------------------------**

**sp|P22748|CAH4_HUMAN --------------------------**

**sp|Q16790|CAH9_HUMAN RRQHRRGTKGGVSYRPAEVAETGA--**

**sp|O43570|CAH12_HUMAN SIKKG--DNKGVIYKPATKMETEAHA**

**sp|Q9ULX7|CAH14_HUMAN IRKKRLENRKSVVFTSAQA-TTEA--**

**Fig. S4** Multiple sequence analysis (MSA) of W7JAI7 selected from group 1 of transmembrane α-CAs of *Plasmodium* spp. with human transmembrane α-CAs.

**tr|Q8IHW5|Q8IHW5_PLAF7 MKLLYLLYPILLFYNVNVFINYKKSRLMLEMIDKYNTHFVQTTKPYYEFNVTNLTNSKKK**

**sp|P22748|CAH4_HUMAN ------------------------------------------------------------**

**sp|Q16790|CAH9_HUMAN ------MAPLCPSPWLPLLIPAPAPGLTVQLL----------------LSLLLLVPVHPQ**

**sp|O43570|CAH12_HUMAN ------------------------------------------------------------**

**sp|Q9ULX7|CAH14_HUMAN ------------------------------------------------------------**

**tr|Q8IHW5|Q8IHW5_PLAF7 KKKKKRENHLIGSGENMQKKDEKNIKDFHINDYEIDGKTIHNKENKDSFKMNKNKLNDNE**

**sp|P22748|CAH4_HUMAN ------------------------------------------------------------**

**sp|Q16790|CAH9_HUMAN RLPRMQEDSPLGGGSSGED-DPLGEEDLPSEE---D--SPREE-----DPPGEEDLPGEE**

**sp|O43570|CAH12_HUMAN ------------------------------------------------------------**

**sp|Q9ULX7|CAH14_HUMAN ------------------------------------------------------------**

**tr|Q8IHW5|Q8IHW5_PLAF7 ELFYMDNILSYKPNK-----KKLFTYSFSENEGNSEKEETLYNFKNMKNINSVQNNINKT**

**sp|P22748|CAH4_HUMAN -------------------MRMLLALLALS-------------------AARPSASAESH**

**sp|Q16790|CAH9_HUMAN DLPGEEDLPEVKPKSEEEGSLKLEDLPTVEAPGDPQEPQN-------NAHRDKEGDDQSH**

**sp|O43570|CAH12_HUMAN -----------MPRRSLHAAAVLLLVILKEQ------------------PSSPAPVNGSK**

**sp|Q9ULX7|CAH14_HUMAN ---------------------MLFSALLLEV------------------IWILAADGGQH**

*** . .**

**tr|Q8IHW5|Q8IHW5_PLAF7 FLYNKLKNVDYYEHGYNWDIGQCKTGKYQSPVDLPMKDLKERELKNISDVYLNLFDDDNY**

**sp|P22748|CAH4_HUMAN WCYEVQAESSNYPCLVPVKWGGNCQKDRQSPINIVTTKAKVDK--K-----LG-----RF**

**sp|Q16790|CAH9_HUMAN WRYG---GDPPWPRVSP-----ACAGRFQSPVDIRPQLAAFCP--A-----LR-----PL**

**sp|O43570|CAH12_HUMAN WTYFGPDGENSWSKKYP-----SCGGLLQSPIDLHSDILQYDA--S-----LT-----PL**

**sp|Q9ULX7|CAH14_HUMAN WTYEGPHGQDHWPASYP-----ECGNNAQSPIDIQTDSVTFDP--D-----LP-----AL**

**: * : ***::: ***

**tr|Q8IHW5|Q8IHW5_PLAF7 AWNNYNKPWMKGDFFYYYEYFIKKIVINRQNNIFQIKAARDGIIPFGVLF-TTEQPAMFY**

**sp|P22748|CAH4_HUMAN FFSGYDKK--------------QTWTV--QNNGHSVM----MLLENKASISGGGLPAPYQ**

**sp|Q16790|CAH9_HUMAN ELLGFQLPPL------------PELRL--RNNGHSVQ----LTLPPGLEM-ALGPGREYR**

**sp|O43570|CAH12_HUMAN EFQGYNLSAN------------KQFLL--TNNGHSVK----LNLPSDMHI-Q-GLQSRYS**

**sp|Q9ULX7|CAH14_HUMAN QPHGYDQPGT------------EPLDL--HNNGHTVQ----LSLPSTLYL-G-GLPRKYV**

**:: : ** . : : : :**

**tr|Q8IHW5|Q8IHW5_PLAF7 ADQIHFHAP-------SEHTFQGSGNRREIEMQIFHSTNYFYDIQDDKSKYKKKYGLHIY**

**sp|P22748|CAH4_HUMAN AKQLHLHWSDLPYK-GSEHSLDGE--HFAMEMHIVHEKEKGTSRNVKE-AQDPEDEIAVL**

**sp|Q16790|CAH9_HUMAN ALQLHLHWGAAGRP-GSEHTVEGH--RFPAEIHVVHLST-AFA-RVDE-ALGRPGGLAVL**

**sp|O43570|CAH12_HUMAN ATQLHLHWGNPNDPHGSEHTVSGQ--HFAAELHIVHYNSDLYP-DAST-ASNKSEGLAVL**

**sp|Q9ULX7|CAH14_HUMAN AAQLHLHWGQKGSPGGSEHQINSE--ATFAELHIVHYDSDSYD-SLSE-AAERPQGLAVL**

*** *:*:* *** ... *:::.* . : :**

HXH

H

**tr|Q8IHW5|Q8IHW5_PLAF7 NNLKKNSKETSKKDSSRYHSYLMSFLMNSLSNEQLQNKYNKKKRIKKMKNQYEVISITFT**

**sp|P22748|CAH4_HUMAN -----------------------AFLVEA-------------------------------**

**sp|Q16790|CAH9_HUMAN -----------------------AAFLEE-------------------------------**

**sp|O43570|CAH12_HUMAN -----------------------AVLIEM-------------------------------**

**sp|Q9ULX7|CAH14_HUMAN -----------------------GILIEV-------------------------------**

**. :::**

**tr|Q8IHW5|Q8IHW5_PLAF7 SAEINASTINAFKKLPSEKFLRTIINVSSAVHVGSDPTLVELKDALNLDALMMMLNIEDM**

**sp|P22748|CAH4_HUMAN ----GTQVNEGFQP-----LVEALSN---IPKPEMSTTM----AESSLLDLLP-KEEKLR**

**sp|Q16790|CAH9_HUMAN ----GPEENSAYEQ-----LLSRLEE---IAEEGSETQV----PGLDISALLP-S--DFS**

**sp|O43570|CAH12_HUMAN ----GSF-NPSYDK-----IFSHLQH---VKYKGQEAFV----PGFNIEELLP-E--RTA**

**sp|Q9ULX7|CAH14_HUMAN ----GETKNIAYEH-----ILSHLHE---VRHKDQKTSV----PPFNLRELLP-K--QLG**

**.:. :. : . . : .: *:**

**tr|Q8IHW5|Q8IHW5_PLAF7 QFLSYQGSSTLPLCDENVSWKVAKQPLPVSTETILNFYYLLKKHTP--NYSGSDNDNYRS**

**sp|P22748|CAH4_HUMAN HYFRYLGSLTTPTCDEKVVWTVFREPIQLHREQILAFSQKLYYDKE---QTVSMKDNVRP**

**sp|Q16790|CAH9_HUMAN RYFQYEGSLTTPPCAQGVIWTVFNQTVMLSAKQLHTLSDTLWGPG-----DSRLQLNFRA**

**sp|O43570|CAH12_HUMAN EYYRYRGSLTTPPCNPTVLWTVFRNPVQISQEQLLALETALYCTHMDDPSPREMINNFRQ**

**sp|Q9ULX7|CAH14_HUMAN QYFRYNGSLTTPPCYQSVLWTVFYRRSQISMEQLEKLQGTLFSTEE--EPSKLLVQNYRA**

**.: * ** * * * * *.* . : : : : * * ***

**tr|Q8IHW5|Q8IHW5_PLAF7 LQNVEDNTRHYRKFSLVQVFPIQVLISSAISNIEDKKVINIIKDISPKNMSFTYYSKWDI**

**sp|P22748|CAH4_HUMAN LQQLGQRT----------------VIKSGAPGR-------------PL--------PWAL**

**sp|Q16790|CAH9_HUMAN TQPLNGRV----------------IEASFPAGVDSSP-----RAAEPVQ----LNSCLAA**

**sp|O43570|CAH12_HUMAN VQKFDERL----------------VYTSFSQV----------------------QVCTAA**

**sp|Q9ULX7|CAH14_HUMAN LQPLNQRM----------------VFASFIQA----------------------GSSYTT**

*** . . : ***

**tr|Q8IHW5|Q8IHW5_PLAF7 YF-------I-------LFIFYNIVLFLF-----------------------------**

**sp|P22748|CAH4_HUMAN PALLGPMLACLLAGFLR-----------------------------------------**

**sp|Q16790|CAH9_HUMAN G----DILALVF-----GLLFAVTSVAFLVQMRRQHRRGTKGGVSYRPAEVAETGA--**

**sp|O43570|CAH12_HUMAN GLSLGIILSLALAGILGICIVVVVSIWLFRRKSIKKG--DNKGVIYKPATKMETEAHA**

**sp|Q9ULX7|CAH14_HUMAN G----EMLSLGV-GILVGCLCLLLAVYFIARKIRKKRLENRKSVVFTSAQA-TTEA—**

**Fig. S5** Multiple sequence analysis (MSA) of Q8IHW5 selected from group 2 of transmembrane α-CAs of *Plasmodium* spp. with human transmembrane α-CAs.

**tr|V7PFH4|V7PFH4_9APIC MKHIIFLSIVLCFCDNVMYNNYVERMLFELPNNITDDLNSDPIVEYKIKEKKNDNIDINK**

**sp|P22748|CAH4_HUMAN ------------------------------------------------------------**

**sp|Q16790|CAH9_HUMAN ---------MAPLCPSP-------------------------WLPLLIPAPA-PGLTVQL**

**sp|O43570|CAH12_HUMAN ------------------------------------------------------------**

**sp|Q9ULX7|CAH14_HUMAN ------------------------------------------------------------**

**tr|V7PFH4|V7PFH4_9APIC DVRHWDIEINE-HKDDPNIQRNIEWHDNNDGNGNNNDNNNGNNSGNNSGNNNGNNSGNNN**

**sp|P22748|CAH4_HUMAN ------------------------------------------------------------**

**sp|Q16790|CAH9_HUMAN LLSL-LLLVPVHPQRLPRMQED-----SPLGGGS---------SG----EDD--------**

**sp|O43570|CAH12_HUMAN ------------------------------------------------------------**

**sp|Q9ULX7|CAH14_HUMAN ------------------------------------------------------------**

**tr|V7PFH4|V7PFH4_9APIC DDNNDNDYGNDKNWEYNSNYNDEEFERQNENERDEFSLKNEVEKNSEERKERAFDESNEY**

**sp|P22748|CAH4_HUMAN ------------------------------------------------------------**

**sp|Q16790|CAH9_HUMAN -PLGEEDLPSEE-----------DSPR-EEDPPGEEDLPGEEDLPGEE----DLPEVKPK**

**sp|O43570|CAH12_HUMAN ---------------------------------------------------------MPR**

**sp|Q9ULX7|CAH14_HUMAN ------------------------------------------------------------**

**tr|V7PFH4|V7PFH4_9APIC ADFENMNDLENMNNIEKEKKKYFEDMQSKYHQQNEYEQNEHQQNEYEQNEHQQNEYEQNE**

**sp|P22748|CAH4_HUMAN -----MRMLLALLALS----------------------------------AARPSASAES**

**sp|Q16790|CAH9_HUMAN SEEEGSLKLEDLPTVEAP---------------------GDP-QEPQNNAHRDKEGDDQS**

**sp|O43570|CAH12_HUMAN RSLHAAAVLLLVILKEQ---------------------------------PSSPAPVNGS**

**sp|Q9ULX7|CAH14_HUMAN -------MLFSALLLEV---------------------------------IWILAADGGQ**

*** . .**

**tr|V7PFH4|V7PFH4_9APIC HQQYEQNEQNEEGNIKNGMIQNNENLSFNYAKHGMDWNVGICKNGKYQSPVDLHMHTLKE**

**sp|P22748|CAH4_HUMAN HWCYEVQAE-------------SSNYP---CLVPVKW--GGNCQKDRQSPINIVTTKAKV**

**sp|Q16790|CAH9_HUMAN HWRYG---G-------------DPPWP---RVSP-------ACAGRFQSPVDIRPQLAAF**

**sp|O43570|CAH12_HUMAN KWTYFGPDG-------------ENSWS---KKYP-------SCGGLLQSPIDLHSDILQY**

**sp|Q9ULX7|CAH14_HUMAN HWTYEGPHG-------------QDHWP---ASYP-------ECGNNAQSPIDIQTDSVTF**

**: * . ***:::**

**tr|V7PFH4|V7PFH4_9APIC RELKNLSDFYLNAFYDNDEYSWNNFNRPWFKGDIFYYYENLINKIIINRQNNMFKIKASN**

**sp|P22748|CAH4_HUMAN D--KKLGRFFFSGYDKK--QTWTV-------------------------QNNGHSVM---**

**sp|Q16790|CAH9_HUMAN C--PALRPLELLGFQLPPLPELRL-------------------------RNNGHSVQ---**

**sp|O43570|CAH12_HUMAN D--ASLTPLEFQGYNLSANKQFLL-------------------------TNNGHSVK---**

**sp|Q9ULX7|CAH14_HUMAN D--PDLPALQPHGYDQPGTEPLDL-------------------------HNNGHTVQ---**

*** : .: ** ..:**

**tr|V7PFH4|V7PFH4_9APIC NEIIPFGVLFTT-DEPTIFYSHHIN-------FHSPSEHTFEGSGNRRHIEMQIYHSTNE**

**sp|P22748|CAH4_HUMAN -MLLENKASISGGGLPAPYQAKQLHLHWSDLPYK-GSEHSLDG--EHFAMEMHIVHEKEK**

**sp|Q16790|CAH9_HUMAN -LTLPPGLEM-ALGPGREYRALQLHLHWGAAGRP-GSEHTVEG--HRFPAEIHVVHLST-**

**sp|O43570|CAH12_HUMAN -LNLPSDMHI-Q-GLQSRYSATQLHLHWGNPNDPHGSEHTVSG--QHFAAELHIVHYNSD**

**sp|Q9ULX7|CAH14_HUMAN -LSLPSTLYL-G-GLPRKYVAAQLHLHWGQKGSPGGSEHQINS--EATFAELHIVHYDSD**

**: : : : ::. *** ... . *::: ***

HXH

H

**tr|V7PFH4|V7PFH4_9APIC IYDYDENKWNGVFEKKNYKKKNNETNIQHSYILTFLMNSLSNPHLGQQYTKNKKRNKRSK**

**sp|P22748|CAH4_HUMAN GTSRNVKEA---------------------------------------------------**

**sp|Q16790|CAH9_HUMAN AFA-RVDEA---------------------------------------------------**

**sp|O43570|CAH12_HUMAN LYP-DASTA---------------------------------------------------**

**sp|Q9ULX7|CAH14_HUMAN SYD-SLSEA---------------------------------------------------**

**.**

**tr|V7PFH4|V7PFH4_9APIC SLYNSIRLDENGKNTKRENQYQVISITFSSAEIDKSTINNFKKLPSEKFLKTILEASQNV**

**sp|P22748|CAH4_HUMAN --------------QDPEDEIAVLAFLVEAGTQVN---EGF---------QPLVEALSNI**

**sp|Q16790|CAH9_HUMAN --------------LGRPGGLAVLAAFLEEGPEEN---SAY---------EQLLSRLEEI**

**sp|O43570|CAH12_HUMAN --------------SNKSEGLAVLAVLIEMGSF-N---PSY---------DKIFSHLQHV**

**sp|Q9ULX7|CAH14_HUMAN --------------AERPQGLAVLGILIEVGETKN---IAY---------EHILSHLHEV**

***:. .. . : : . :.. .:**

**tr|V7PFH4|V7PFH4_9APIC PVGSDPKLVNLKEPLNLNSLLMMLNMKSMEFFAYHGSSTSPDCNENVHWKVAKKSLPIST**

**sp|P22748|CAH4_HUMAN PKPEMS---TTMAESSLLDLL-PKEEKLRHYFRYLGSLTTPTCDEKVVWTVFREPIQLHR**

**sp|Q16790|CAH9_HUMAN AEEGSE---TQVPGLDISALL-PS--DFSRYFQYEGSLTTPPCAQGVIWTVFNQTVMLSA**

**sp|O43570|CAH12_HUMAN KYKGQE---AFVPGFNIEELL-PE--RTAEYYRYRGSLTTPPCNPTVLWTVFRNPVQISQ**

**sp|Q9ULX7|CAH14_HUMAN RHKDQK---TSVPPFNLRELL-PK--QLGQYFRYNGSLTTPPCYQSVLWTVFYRRSQISM**

**.: ** .:: * ** *:* * * *.* . :**

**tr|V7PFH4|V7PFH4_9APIC ETMLKFYNMLKKTTPD--YNASDNDNFRALQNVQGNIHNYGRVYLIQGFPVQLLISSALT**

**sp|P22748|CAH4_HUMAN EQILAFSQKLYYDKE---QTVSMKDNVRPLQQLGQRTVI-------KSGAPGR-------**

**sp|Q16790|CAH9_HUMAN KQLHTLSDTLWGPG-----DSRLQLNFRATQPLNGRVIE-------ASFPAGVDSSPRA-**

**sp|O43570|CAH12_HUMAN EQLLALETALYCTHMDDPSPREMINNFRQVQKFDERLVY-------TSFSQV--------**

**sp|Q9ULX7|CAH14_HUMAN EQLEKLQGTLFSTEE--EPSKLLVQNYRALQPLNQRMVF-------ASFIQA--------**

**: : : * * * * . . .**

**tr|V7PFH4|V7PFH4_9APIC TSEDKNVIENIKLAYSKSSGNYIYF-------NLIFLLLIFIFLQNY-------------**

**sp|P22748|CAH4_HUMAN -----PL----PWALPALLGPMLACLLAGFLR----------------------------**

**sp|Q16790|CAH9_HUMAN ---AEPVQLNSCLAAG----DILALVF-----GLLFAVTSVAFLVQMRRQHRRGTKGGVS**

**sp|O43570|CAH12_HUMAN ---------QVCTAAGLSLGIILSLALAGILGICIVVVVSIWLFRRKSIKKG--DNKGVI**

**sp|Q9ULX7|CAH14_HUMAN ---------GSSYTTG----EMLSLGV-GILVGCLCLLLAVYFIARKIRKKRLENRKSVV**

**: :**

**tr|V7PFH4|V7PFH4_9APIC -------------**

**sp|P22748|CAH4_HUMAN -------------**

**sp|Q16790|CAH9_HUMAN YRPAEVAETGA--**

**sp|O43570|CAH12_HUMAN YKPATKMETEAHA**

**sp|Q9ULX7|CAH14_HUMAN FTSAQA-TTEA--**

**Fig. S6** Multiple sequence analysis (MSA) containing transmembrane η-CA (V7PFH4) of *Plasmodium* spp. and human transmembrane α-CAs.

**
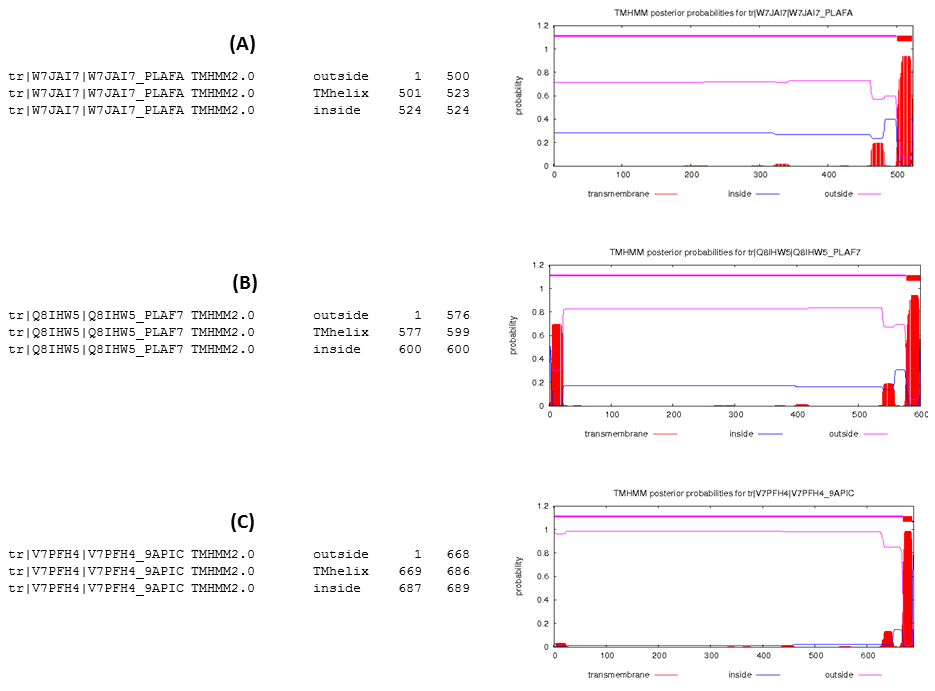
**

**Fig. S7 Prediction of transmembrane localization of representative α- and η-CAs from *Plasmodium* spp.** The prediction identified transmembrane segments near the C-terminal ends of W7JAI7 from group 1 (A) and Q8IHW5 from group 2 (B) of *P. falciparum* α-CAs and V7PFH4 from *P. yoelii* η-CAs (C).

2W2J

W7JA MQKKDEKNIKDFHINDYEIDGKTIHNKENKDSFKMNKNKLNDNEELFYMDNILSYKPNKK

. :* ::::

2W2J EEE--------------------------GVEWGYEEGVEWGLV

W7JA KLFTYSFSENEGNSEKEETLYNFKNMKNINSVQNNINKTFLYNKLKNVDYYEHGYNWDIG

:* *:* :.*:** **.* :* :

2W2J FPDANGEYQSPINLNSREARYDP--SLLDVRL----SPNYVVC-----------------

W7JA -QCKTGKYQSPVDLPMKDLKERELKNISDVYLNLFDDDNYAWNNYNKPWMKGDFFYYYEY

.*:****::* :: : .: ** * . **.

2W2J --RDCEVTNDGHTIQVILKSKSVLSGGPLPQGHEFE------LYEVRFHWGREN------

W7JA FIKKIVINRQNNIFQIK-----AARDGIIPFGVLFTTEQPAMFYADQIHFHAPSEHTFQG

:. :..: . :*: . * :* * * :* ::*: .

2W2J ---QRG------------------------------------------SEHTVNFKAFPM

W7JA SGNRREIEMQIFHSTNYFYDIQDDKSKYKKKYGLHIYNNLKKNSKETSKKDSSRYHSYLM

:* .:.: .:::: *

2W2J ELHLIHWNSTLFGSIDEAVGKPHGI---------------------AIIALFVQIGKEHV

W7JA SFLMNSLSN---EQLQNKYNKKKRIKKMKNQYEVISITFTSAEINASTINAFKKLPSEKF

.: : .. .::: * : * : * * :: .*:.

2W2J GLKAVTEILQDIQYKGKSKTIP----CFN--PNTLLPDPLLRDYWVYEGSLTIPPCSEGV

W7JA -LRTIINVSSAVH-VGSDPTLVELKDALNLDALMMMLNIEDMQFLSYQGS**STLPLCDENV**

*::: :: . :: *.. *: .:* :: : :: *:** *:* *.* *

2W2J TWILFRYPLTISQLQIEEFRRLRTHVKGAELVEGCDGILGDNFRPTQPLSDRVIRA----

W7JA **SWK**VAKQPLPVSTETILNFYYLLKKH-----TPNYSGSDNDNYRSLQNVEDNTRHY**RKFS**

:*: : ** :* * :* * .: . .* **:* * :.*.. :

2W2J ---AFQ

W7JA **LVQVFPIQVLISS**AISNIEDKKVINIIKDISPKNMSFSYYSKWDIYFILFIFYNIVLFLF

.*

**Fig. S8** Pairwise sequence alignment of PDB 2W2J and W7JAI7 V7PFH4 indicating the locations of predicted MHC-I ligands.

4XIW AWNYGE

Q8IW ELFYMDNILSYKPNKKKLFTYSFSENEGNSEKEETLYNFKNMKNINSVQNNINKTFLYNK

:: * :

4XIW V-------AGPPTWKGVCATGKRQSPINIPLNTSAPKVDAE-------------------

Q8IW LKNVDYYEHGYNWDIGQCKTGKYQSPVDLPMKDLKERELKNISDVYLNLFDDDNYAWNNY

: * * * *** ***:::*:: : :

4XIW -----MGEFDFAYGSFEKCDVLNTGHGTMQVNFPAGNLAFIG-------NMELELLQFHF

Q8IW NKPWMKGDFFYYYEYFIKKIVINRQNNIFQIKAARDGIIPFGVLFTTEQPAMFYADQIHF

*:* : * * * *:* . :*:: : :* : *:**

4XIW HAPSEHAMDGRRYAME--AHLVHKNKS-----------------------------T---

Q8IW HAPSEHTFQGSGNRREIEMQIFHSTNYFYDIQDDKSKYKKKYGLHIYNNLKKNSKETSKK

******:::* * ::.*..: *

4XIW ------------------------------------GNLAVLGIMLEPGGL---------

Q8IW DSSRYHSYLMSFLMNSLSNEQLQNKYNKKKRIKKMKNQYEVISITFTSAEINASTINAFK

: *:.* : . :

4XIW --IKNPALSTALEVAPEVPLAKKPSP------KGINPVMLLPKKSKAGTRPFVHYPGSLT

Q8IW KLPSEKFLRTIINVSSAVHVGSDPTLVELKDALNLDALMM---MLNIEDMQFLSYQG**SST**

.: * * ::*: * :...*: :: :*: : *: * ** *

4XIW TPPCSEGVDWFVFMQPIKVPDSQILDFMRFVGDNKTYAT-----NTRPLQLLNSRLVEYE

Q8IW **LPLCDENVSWK**VAKQPLPVSTETILNFYYLLKKHTPNYSGSDNDNYRSLQNVEDNTRHYR

* *.* *.* * **: * . **:* :: ... : * * ** ::.. .*.

4XIW L

Q8IW KFSLVQVFPIQVLISSAISNIEDKKVINIIKDISPKNMSFTYYSKWDIYFILFIFYNIVL

4XIW

Q8IW FLF

**Fig. S9** Pairwise sequence alignment of PDB 3FE4 and Q8IHW5 indicating the locations of predicted MHC-I ligands.

3FE4 eahwpqhypacggqrqspinlqrtk

V7PF QQYEQNEQNEEGNIKNGMIQNNENLSFNYAKH---GMDWNVGI-CKNGKYQSPVDLHMHT

.:.*:: .* . *: ***::*: .

3FE4 vry--------------------------npslkglnmtgyetqagefpmvnngh-----

V7PF LKERELKNLSDFYLNAFYDNDEYSWNNFNRPWFKGDIFYYYENLINKIIINRQNNMFKIK

:: .* :** : **. :: : .: .

3FE4 -tvqislpstmrmtvadgtvyiaqqmhfhwggasseisgsehtvdgirhvieihivhyns

V7PF ASNNEIIPFGVLFTTDEPTIFYSHHINFHS--------PSEHTFEGSGNRRHIEMQIYHS

: : :* : :*. : *:: ::::.** ****.:* . .*.: *.*

3FE4 kyksydiaqdapdg----------------------------------------------

V7PF TNEIYDYDENKWNGVFEKKNYKKKNNETNIQHSYILTFLMNSLSNPHLGQQYTKNKKRNK

. : ** :: :*

3FE4 -----------------------lav-----------laafvevknypentyysnfishl

V7PF RSKSLYNSIRLDENGKNTKRENQYQVISITFSSAEIDKSTINNFKKLPSEKFLKTILEAS

* ::: :.*: *.:.: ..::.

3FE4 anikyp------gqrttltgldvqdmlprnlqhyytyhgslttppctenvhwfvladfvk

V7PF QNVPVGSDPKLVNLKEPLNLNSLLMMLNMKSMEFFAYHGSSTSPDCNENVHWKVAKKSLP

*: : *. .: ** : .:::**** *:* *.***** * . :

3FE4 lsrtqvwklenslldhrnk---tihndyrrtqplnhr-------vvesnfpn

V7PF ISTETMLKFYNMLKKTTPDYNASDNDNFRALQNVQGNIH**NYGRVYLIQGFPVQLLISSAL**

:* : *: * * . . : .:::* * :: . : . ** * .:

3FE4

V7PF TTSEDKNVIENIKLAYSKSSGNYIYFNLIFLLLIFIFLQNY

**Fig. S10** Pairwise sequence alignment of PDB 3FE4 and V7PFH4 indicating the locations of predicted MHC-I ligands.

2W2J

W7JA MQKKDEKNIKDFHINDYEIDGKTIHNKENKDSFKMNKNKLNDNEELFYMDNILSYKPNKK

. :* ::::

2W2J EEE--------------------------GVEWGYEEGVEWGLV

W7JA KLFTYSFSENEGNSEKEETLYNFKNMKNINSVQNNINKTFLYNKLKNVDYYEHGYNWDIG

:* *:* :.*:** **.* :* :

2W2J FPDANGEYQSPINLNSREARYDP--SLLDVRL----SPNYVVC-----------------

W7JA -QCKTGKYQSPVDLPMKDLKERELKNISDVYLNLFDDDNYAWNNYNKPWMKGDFFYYYEY

.*:****::* :: : .: ** * . **.

2W2J --RDCEVTNDGHTIQVILKSKSVLSGGPLPQGHEFE------LYEVRFHWGREN------

W7JA FIKKIVINRQNNIFQIK-----AARDGIIPFGVLFTTEQPAMFYADQIHFHAPSEHTFQG

:. :..: . :*: . * :* * * :* ::*: .

2W2J ---QRG------------------------------------------SEHTVNFKAFPM

W7JA SGNRREIEMQIFHSTNYFYDIQDDKSKYKKKYGLHIYNNLKKNSKETSKKDSSRYHSYLM

:* .:.: .:::: *

2W2J ELHLIHWNSTLFGSIDEAVGKPHGI---------------------AIIALFVQIGKEHV

W7JA SFLMNSLSN---EQLQNKYNKKKRIKKMKNQYEVISITFTSAEINASTINAFKKLPSEKF

.: : .. .::: * : * : * * :: .*:.

2W2J GLKAVTEILQDIQYKGKSKTIP----CFN--PNTLLPDPLLRDYWVYEGSLTIPPCSEGV

W7JA -**LRTIINVSSAVH**-**VGSDPTLVELK**DALNLDALMMMLNIEDMQFLSYQGSSTLPLCDENV

*::: :: . :: *.. *: .:* :: : :: *:** *:* *.* *

2W2J TWILFRYPLTISQLQIEEFRRLRTHVKGAELVEGCDGILGDNFRPTQPLSDRVIRA----

W7JA SWKVAKQPLPVSTETILNFYYLLKKH-----TPNYSGSDNDNYRSLQNVEDNTRHY**RKFS**

:* : : ** :* * :* * .: . .* **:* * :.*.. :

2W2J ---AFQ

W7JA **LVQVFPIQVLISS**AISNIEDKKVINIIKDISPKNMSFSYYSKWDIYFILFIFYNIVLFLF

.*

**Fig. S11** Pairwise sequence alignment of PDB 2W2J and **W7JAI7** indicating the locations of predicted MHC-II ligands.

4XIW AWNYGE

Q8IW ELFYMDNILSYKPNKKKLFTYSFSENEGNSEKEETLYNFKNMKNINSVQNNINKTFLYNK

:: * :

4XIW V-------AGPPTWKGVCATGKRQSPINIPLNTSAPKVDAE-------------------

Q8IW LKNVDYYEHGYNWDIGQCKTGKYQSPVDLPMKDLKERELKNISDVYLNLFDDDNYAWNNY

: * * * *** ***:::*:: : :

4XIW -----MGEFDFAYGSFEKCDVLNTGHGTMQVNFPAGNLAFIG-------NMELELLQFHF

Q8IW NKPWMKGDFFYYYEYFIKKIVINRQNNIFQIKAARDGIIPFGVLFTTEQPAMFYADQIHF

*:* : * * * *:* . :*:: : :* : *:**

4XIW HAPSEHAMDGRRYAME--AHLVHKNKS-----------------------------T---

Q8IW HAPSEHTFQGSGNRREIEMQIFHSTNYFYDIQDDKSKYKKKYGLHIYNNLKKNSKETSKK

******:::* * ::.*..: *

4XIW ------------------------------------GNLAVLGIMLEPGGL---------

Q8IW DSSRYHSYLMSFLMNSLSNEQLQNKYNKKKRIKKMKNQYEVISITFTSAEINASTINAFK

: *:.* : . :

4XIW --IKNPALSTALEVAPEVPLAKKPSP------KGINPVMLLPKKSKAGTRPFVHYPGSLT

Q8IW KLP**SEKFLRTIINVSSAVHVGSDPTLVELK**DALNLDALMM---MLNIEDMQFLSYQGSST

.: * * ::*: * :...*: :: :*: : *: * ** *

4XIW TPPCSEGVDWFVFMQPIKVPDSQILDFMRFVGDNKTYAT-----NTRPLQLLNSRLVEYE

Q8IW LPLCDENVSWKVAKQPLPVSTETILNFYYLLKKHTPNYSGSDNDNYRSLQNVEDNTRHYR

* *.* *.* * **: * . **:* :: ... : * * ** ::.. .*.

4XIW L

Q8IW KFSLVQVFPIQVLISSAISNIEDKKVINIIKDISPKNMSFTYYSKWDIYFILFIFYNIVL

4XIW

Q8IW FLF

**Fig. S12** Pairwise sequence alignment of PDB PDB 3FE4 and Q8IHW5 indicating the location of predicted MHC-II ligand.

3FE4 eahwpqhypacggqrqspinlqrtk

V7PF QQYEQNEQNEEGNIKNGMIQNNENLSFNYAKH---GMDWNVGI-CKNGKYQSPVDLHMHT

.:.*:: .* . *: ***::*: .

3FE4 vry--------------------------npslkglnmtgyetqagefpmvnngh-----

V7PF LKERELKNLSDFYLNAFYDNDEYSWNNFNRPWFKGDIFYYYENLINKIIINRQNNMFKIK

:: .* :** : **. :: : .: .

3FE4 -tvqislpstmrmtvadgtvyiaqqmhfhwggasseisgsehtvdgirhvieihivhyns

V7PF ASNNEIIPFGVLFTTDEPTIFYSHHINFHS--------PSEHTFEGSGNRRHIEMQIYHS

: : :* : :*. : *:: ::::.** ****.:* . .*.: *.*

3FE4 kyksydiaqdapdg----------------------------------------------

V7PF TNEIYDYDENKWNGVFEKKNYKKKNNETNIQHSYILTFLMNSLSNPHLGQQYTKNKKRNK

. : ** :: :*

3FE4 -----------------------lav-----------laafvevknypentyysnfishl

V7PF RSKSLYNSIRLDENGKNTKRENQYQVISITFSSAEIDKSTINNFKKLPSEKFLKTILEAS

* ::: :.*: *.:.: ..::.

3FE4 anikyp------gqrttltgldvqdmlprnlqhyytyhgslttppctenvhwfvladfvk

V7PF QNVPVGSDPKLVNLKEPLNLNSLLMMLNMKSMEFFAYHGSSTSPDCNENVHWKVAKKSLP

*: : *. .: ** : .:::**** *:* *.***** * . :

3FE4 lsrtqvwklenslldhrnk---tihndyrrtqplnhr-------vvesnfpn

V7PF ISTETMLKFYNMLKKTTPDYNASDNDNFRALQNVQGNIHN**YGRVYLIQGFPVQLLISSAL**

:* : *: * * . . : .:::* * :: . : . ** * .:

3FE4

V7PF **T**TSEDKNVIENIKLAYSKSSGNYIYFNLIFLLLIFIFLQNY

**Fig. S13** Pairwise sequence alignment of PDB 3FE4 and V7PFH4 indicating the location of predicted MHC-II ligand.
