# Supplementary material for: A reverse vaccinology approach on transmembrane carbonic anhydrases from Plasmodium species as vaccine candidates for malaria prevention
Source: Malar J. 2022 Jun 15;21:189. doi: 10.1186/s12936-022-04186-7 (PMC9199335; doi:10.1186/s12936-022-04186-7)
Supplement: Supplementary file 2 — Additional file 2: Table S1. Disulfide-bond prediction of Plasmodium tmCAs. [file 12936_2022_4186_MOESM2_ESM.docx]

**Table S1 Disulfide-bond prediction of *Plasmodium* tmCAs**

| **CA class** | **Total number of disulfide-bonded Cys residues** | **Location of disulfide-bonded Cys residues** | **Conn_conf^+^** | **Supported by homology modelling** |
| --- | --- | --- | --- | --- |
| **α-CA**  **(group 1)**  **(W7JAI7)** | 2 | Cys122 | 1 for Cys122= Cys405 | Yes, proximal |
|  |  | Cys405 |  |  |
| **α-CA**  **(group 2)**  **(Q8IHW5)** | 2 | Cys198 | 1 for Cys198= Cys481 | Yes, disulfide bonded |
|  |  | Cys481 | 1 for Cys198= Cys481 |  |
| **η-CA**  **(V7PFH4)** | 2 | Cys12 | 0.8 for Cys12= Cys14 | N/A |
|  |  | Cys14 |  |  |
|  |  | Cys281 | 0.8 for Cys281= Cys574 | N/A |
|  |  | Cys574 |  |  |

**+: Conn_conf:** confidence of connectivity assignment given the predicted disulfide bonding state (real value in [0,1]).
